# Comparison of *Xenorhabdus bovienii* bacterial strain genomes reveals diversity in symbiotic functions

**DOI:** 10.1186/s12864-015-2000-8

**Published:** 2015-11-02

**Authors:** Kristen E. Murfin, Amy C. Whooley, Jonathan L. Klassen, Heidi Goodrich-Blair

**Affiliations:** Department of Bacteriology, University of Wisconsin-Madison, Madison, WI 53706 USA; Department of Molecular & Cell Biology, University of Connecticut, Storrs, CT 06269 USA

**Keywords:** Symbiosis, Competition, *Xenorhabdus*, *Steinernema*, Bacteria, Nematodes, Insects, Comparative genomics

## Abstract

**Background:**

*Xenorhabdus* bacteria engage in a beneficial symbiosis with *Steinernema* nematodes, in part by providing activities that help kill and degrade insect hosts for nutrition. *Xenorhabdus* strains (members of a single species) can display wide variation in host-interaction phenotypes and genetic potential indicating that strains may differ in their encoded symbiosis factors, including secreted metabolites.

**Methods:**

To discern strain-level variation among symbiosis factors, and facilitate the identification of novel compounds, we performed a comparative analysis of the genomes of 10 *Xenorhabdus bovienii* bacterial strains.

**Results:**

The analyzed *X. bovienii* draft genomes are broadly similar in structure (e.g. size, GC content, number of coding sequences). Genome content analysis revealed that general classes of putative host-microbe interaction functions, such as secretion systems and toxin classes, were identified in all bacterial strains. In contrast, we observed diversity of individual genes within families (e.g. non-ribosomal peptide synthetase clusters and insecticidal toxin components), indicating the specific molecules secreted by each strain can vary. Additionally, phenotypic analysis indicates that regulation of activities (e.g. enzymes and motility) differs among strains.

**Conclusions:**

The analyses presented here demonstrate that while general mechanisms by which *X. bovienii* bacterial strains interact with their invertebrate hosts are similar, the specific molecules mediating these interactions differ. Our data support that adaptation of individual bacterial strains to distinct hosts or niches has occurred. For example, diverse metabolic profiles among bacterial symbionts may have been selected by dissimilarities in nutritional requirements of their different nematode hosts. Similarly, factors involved in parasitism (e.g. immune suppression and microbial competition factors), likely differ based on evolution in response to naturally encountered organisms, such as insect hosts, competitors, predators or pathogens. This study provides insight into effectors of a symbiotic lifestyle, and also highlights that when mining *Xenorhabdus* species for novel natural products, including antibiotics and insecticidal toxins, analysis of multiple bacterial strains likely will increase the potential for the discovery of novel molecules

**Electronic supplementary material:**

The online version of this article (doi:10.1186/s12864-015-2000-8) contains supplementary material, which is available to authorized users.

## Background

**Fig. 1:**
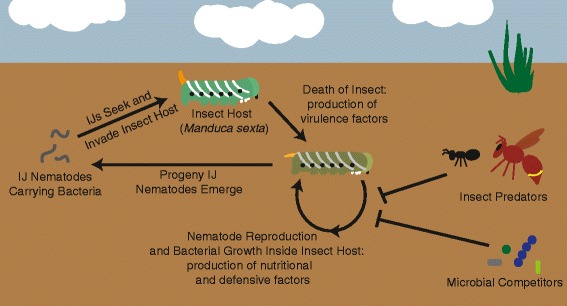
*Xenorhabdus* bacteria and *Steinernema* nematode life cycle. In the soil, *Steinernema* infective juvenile (IJ) nematodes containing their *Xenorhabdus* symbionts seek out and invade insect hosts. Once in the insect blood cavity, the nematodes and bacteria produce virulence factors and kill the insect host. The nematodes and bacteria then grow and reproduce using insect cadaver biomass, a process facilitated by the bacterial symbiont. During reproduction, the growing nematodes and bacteria are vulnerable to insect predators and microbial competitors, and therefore, defensive compounds are produced during reproduction. Once all nutrients within the cadaver are consumed, the nematodes form the next generation of IJs (progeny) that then exit the insect cadaver to seek new insect hosts

*Xenorhabdus* bacteria are beneficial symbionts of entomopathogenic (insect-parasitic) *Steinernema* nematodes. In addition to being effective biocontrol agents for a variety of insect pests [1, 2], *Xenorhabdus – Steinernema* complexes are tractable laboratory systems that facilitate investigation of ecological [3], evolutionary [4, 5] and symbiotic [6, 7] processes. The integrated life cycle of *Xenorhabdus* bacteria and *Steinernema* nematodes comprises alternating environments of the soil and insect hosts infected by the pair (Fig. 1) [6]. The infective juvenile (IJ) is the soil dwelling, environmental stage of the nematode that carries bacteria and infects insect hosts. Once within the insect, the nematodes and bacteria kill the insect and reproduce using the nutrients derived from the cadaver. During reproduction, the nematodes and bacteria are vulnerable to predation by scavenger insects [8, 9] and competition from other opportunistic organisms, such as nematodes, bacteria, or fungi [10–13]. After nutrients within the insect cadaver are consumed, and nematode density is high, the nematodes develop into progeny IJs that exit the cadaver to repeat the cycle [14]. In the association, the bacterial symbiont contributes to virulence against the insect host [5, 15, 16], support of nematode reproduction [4, 5, 17], and defense against encountered competitors, pathogens, and predators [8–10, 18]. In turn, the nematode partner serves as a vector to transmit bacteria between insect hosts [19, 20] and augments bacterial virulence against insects [5, 21, 22].

To accomplish symbiotic functions, *Xenorhabdus* bacteria encode a wide array of bioactive molecules that can serve as virulence factors [23–27], degradative enzymes for nutritional support [17], anti-predatory compounds [8, 9], and anti-microbial compounds [10, 11, 18]. Therefore, *Xenorhabdus* species have been utilized for the identification of novel metabolites with pharmaceutical properties [18, 28, 29], and it has been proposed that other *Xenorhabdus* compounds may be useful for development as insecticides, nematicides, and antimicrobials [18]. Our recent work has revealed intra-species variation (i.e. sub-species by standard metrics [30]) in the ability of *Xenorhabdus bovienii* bacterial strains to engage in symbiosis with *S. feltiae* nematode hosts [5], indicating that examination of multiple strains within a species has the potential to expose additional diversity of bioactive metabolites. Indeed, pan-genomic analysis of *X. bovienii* strains revealed the core genome to be approximately 55 % of total coding content, with 1–9 % of the coding content being unique to an individual strain [5]. The remaining 36–44 % is shared among some, but not all, bacterial strains studied. However, it has not been determined if the bioactive molecules that may contribute to symbiotic functions of *X. bovienii* are conserved or shared. To address this, we performed a comparative genomic analysis on ten previously published bacterial genomes [5, 31, 32] that were isolated from 6 different *Steinernema* spp. nematode hosts (Table 1), focusing on genes and predicted products that are likely to be involved in symbiotic interactions.

**Table 1:** *Xenorhabdus bovienii* genomes used in this study^a^

Genome^a^	*Steinernema* nematode host^b^	Source^c^	Genome Accession^d^ Number^d^	Study^e^	Genome Size (Mb)^f^	G + C content (%)^g^	Number of CDS^h^	Coding Density (%)^i^	Number of Contigs^j^	N50 Value (Kb)^k^
Xb-Sf-FL	*S. feltiae*	Florida, USA	[EMBL:PRJEB432]	5	4.47	44.89	4508	83.43	436	45
Xb-Sf-FR	*S. feltiae*	France	[EMBL:PRJEB4319]	5	4.41	44.75	4441	84.06	449	45
Xb-Sf-MD	*S. feltiae*	Moldova	[EMBL:PRJEB4321]	5	4.66	44.36	4695	83.82	397	52
Xb-Si	*S. intermedium*	North Carolina, USA	[EMBL:PRJEB4327]	5	4.71	44.89	4719	83.39	467	51
Xb-Sj	*S. jollieti*	Monsanto	[EMBL:PRJEB4326]	5	3.93	44.48	3939	83.20	457	46
Xb-Sj-2000	*S. jollieti*	Monsanto	[EMBL:FN667741]	31	4.23	44.97	4406	84.07	1	NA
Xb-Sk-BU	*S. kraussei*	Becker Underwood	[EMBL:PRJEB4325]	5	4.72	44.75	4860	83.10	752	33
Xb-Sk-CA	*S. kraussei*	Quebec, CA	[EMBL:PRJEB4324]	5	4.21	44.18	4097	83.96	422	34
Xb-So	*S. oregonense*	Oregon, USA	[EMBL:PRJEB4323]	5	4.13	44.90	4261	84.30	429	51
Xb-Sp	*S. puntauvense*	Costa Rica	[EMBL:PRJEB4322]	5	4.48	44.32	4584	84.34	443	51

^a^Abbreviation used for bacterial genomes

^b^The *Steinernema* nematode host species from which the *X. bovienii* bacterial strain was isolated

^c^The location or company from which the nematode was obtained

^d^The accession number for the bacterial genome in EMBL

^e^Study in which the genome sequence was originally reported. Number corresponds to the citation

^f^Size of the genome in megabases

^g^GC content percentage of the genome

^h^Total number of coding sequences in the genome

^i^Amount of the genome that is coding sequences

^j^Number of contigs in the genome sequence

^k^Median size of the contigs in megabases. NA indicates not applicable for the finished genome

## Results and discussion

### General genomic features of *Xenorhabdus bovienii*

We recently reported a brief description of draft genomes of 9 *X. bovienii* strains, but did not provide an in depth comparison of the general genomic features relative to the finished *X. bovienii* genome [5, 31, 32]. Here we present a more thorough analysis, which indicated that all the examined *X. bovienii* genomes are similar to each other in genome in size, GC content, number of coding sequences and coding density (Table 1). On average the genomes are 4.4 Mbp, with 4451 coding sequences. No plasmids were detected in any of these strains through sequencing, DNA extraction, or plasmid extraction (data not shown).

**Fig. 2:**
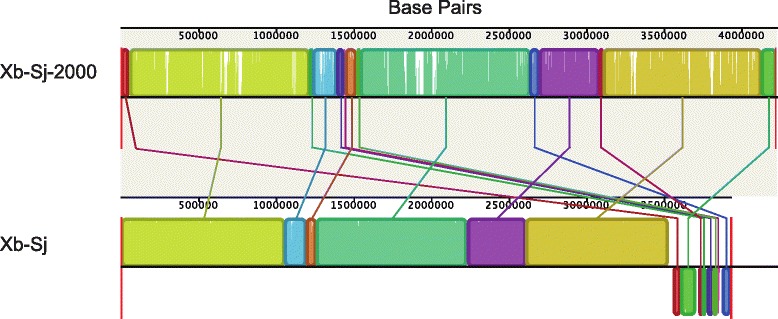
Genome synteny between finished and draft *X. bovienii* genomes. Genomes were aligned with progressive MAUVE to assess the large-scale synteny among the draft *X. bovienii* genomes and the finished *X. bovienii* genome (Xb-Sj-2000). The synteny of the whole length of the genomes is shown. Genome names are indicated on the left, and the scale is shown in base pairs. Colored boxes indicate regions of synteny, and colored lines connect like regions

To assess completeness of the draft genomes, we determined the number and size of contigs. All of the draft genomes have ~400 contigs, with an N50 value between 30 and 55 kilobasepairs (Table 1). Additionally, the draft genome sequence of the *S. jollieti* symbiont (Xb-Sj) shows genome-wide synteny to the finished genome sequence (Xb-Sj-2000), but the draft genome lacks portions found within the complete genome, approximately 0.3Mbp total (Fig. 2). Two regions of the draft Xb-Sj genome that were not associated with the ends of contigs lacked synteny with the complete Xb-Sj-2000 genome (6- and 14-Kb in size), indicating one or more rearrangements in the draft genome. The remaining regions that did not match the draft genome were associated with the ends of contigs, indicating that these breaks may be due to misassembly rather than rearrangements. Xb-Sj and Xb-Sj-2000 are bacterial strains that came from the same nematode host strain but were isolated 7 years apart [15], so the two genomes should be almost identical because these symbionts are transmitted with high fidelity and the nematodes were propagated in the laboratory without exposure to other *Xenorhabdus* species and strains [33]. It is more likely that differences in the size of Xb-Sj and Xb-Sj-2000 genomes is due to genome reduction in the symbiont that had been associated with the host through laboratory propagation for a longer period of time. Additionally, Xb-Sj contains a predicted open reading frame (XBJ2v2_1630007) that is absent from Xb-Sj-2000 based on BLASTn analysis, which indicates that it has gained at least some genetic material in the 7 years between these strains’ isolation. This gene has a GC content lower (17 %) than the remainder of the genome (44 %), suggesting potential horizontal transmission. However, the predicted product is a protein of unknown function and very small in size (69 amino acids) with no significant BLAST analysis hits to proteins or genes of known function.

### Metabolic analysis of *X. bovienii* strains

Previous studies have shown that specific metabolic pathways (e.g. amino acid and vitamin biosynthesis) are integral to some interactions between *Xenorhabdus nematophila* bacterial symbionts and their *Steinernema carpocapsae* host nematodes [34]. To determine if *X. bovienii* bacterial strains differ in metabolic potential, we performed a global metabolic analysis on all 10 bacterial genomes. These analyses found very few differences among the strains (Additional file 1). There were many incomplete pathways, suggesting that annotations may not be entirely accurate. However, all genomes including the complete genome had approximately the same degree of pathway annotation, including biosynthetic pathways, degradative pathways, and energy metabolism. This supports that the absence of some genes within pathways is not due to incomplete draft genome assembly. Of note, the Xb-Sf-MD genome has an incomplete chitin degradation pathway, lacking intact genes to encode chitinase, chitotriose synthase, and diacetylchitobiose synthase, while all other strains have the complete set of genes within this pathway. However, the Xb-Sf-MD genome does contain several pseudogenes (due to early stop codons) with homology to portions of the missing enzymes. Although these bioinformatic data suggest that Xb-Sf-MD may have a deficiency in chitin utilization relative to other *X. bovienii* strains we were unable to verify this experimentally, since under laboratory conditions none of the strains grew on chitin as a sole carbon source (data not shown).

### Secretion systems present in *X. bovienii* strains

For our comparative genomic analyses, we focused on factors likely to contribute to the interaction between *X. bovienii* strains and hosts. Bacterial products that directly interact with host cells must be exported out of the bacterial cell through secretion systems, and disruption of secretion systems in bacterial symbionts can cause defects in pathogenesis [35, 36] and mutualism [37, 38]. To determine which secretion system types are present among *X. bovienii* genomes we searched for gene clusters known to encode systems responsible for secretion of host-interacting factors (Additional file 2). Consistent with published observations [39], none of the *X. bovienii* strains encoded an obvious Type III secretion system, one of the systems most commonly associated with pathogenesis [40]. Furthermore, none of the *X. bovienii* strains encoded a complete Type IV secretion system [41], although various Type IV-system-related genes were present in all of the genomes, as previously noted for the complete genome of *X. bovienii* [39] (data not shown). All *X. bovienii* strains analyzed encode a flagellar export apparatus, which is evolutionarily related to Type III secretion systems. In other bacteria, including *X. nematophila* the flagellar system exports non-flagellar factors such as xenocin (an antibiotic) and virulence determinants [42–44]. All of the *X. bovienii* strains examined here also encode a type II secretion system, which in other Gram-negative bacteria transport folded proteins (e.g. toxins and degradative enzymes) from the periplasm to the extracellular environment [45]. All *X. bovienii* strains analyzed in this study also encode a type VI secretion system. These systems transfer effectors (e.g. hemolysins) directly from the bacterial cell into the target host cell (which can be bacterial or eukaryotic) through injection [46, 47]. Also, each genome contains additional type VI secretion system structural genes, such as multiple *vgrG* genes (data not shown). Finally*,* all of the examined *X. bovienii* strains encode alkaline protease secretion systems, which export the protease PrtA [48]. Overall, the types and numbers of intact secretion systems of *X. bovienii* appear to be conserved, with each genome possessing syntenous regions containing all required secretion system components.

The presence of the same secretion systems (and the absence of others) in all *X. bovienii* strains examined likely reflects their similar symbiotic lifestyles interacting with nematode and insect hosts. However, these bacterial strains associate with divergent nematode host species and likely encounter different insect host species within the environment. Therefore, the secreted effector proteins delivered by the secretion systems are expected to vary. To address this idea, we further investigated bacterial factors, or putative secreted effector molecules, that may be involved in symbiotic interactions.

### Phenotypic testing of *X. bovienii* strains

Some *Xenorhabdus* factors that are likely secreted and involved in symbiosis have readily monitored activities. For example, lipase activity in *X. nematophila* contributes to nutritional support of its nematode host, *Steinernema carpocapsae*, and is secreted through the flagellar apparatus [44, 49, 50]. To assess the diversity of phenotypes associated with symbiosis among the sequenced *X. bovienii* strains, we performed phenotypic testing. We measured activities implicated in bacterial or nematode nutrition, including iron acquisition through siderophore activity [51, 52] and the enzymatic activities of lipase [17, 44], lecithinase [17], and protease [17, 48] that help degrade insect tissues. In addition, we monitored hemolytic activity [25] and motility [49], which are both associated with virulence towards the insect host or subsequent degradation [53–55]. Finally, we assessed antibiotic activity, which likely plays a defensive role in removing competitor or nematode-pathogenic microbes from the insect cadaver [11].

In *Xenorhabdus* species, including *X. bovienii,* assessment of the activities listed above is complicated by phenotypic variation. All *Xenorhabdus* spp. studied to date undergo phenotypic variation between primary and secondary form cells that have differing levels of some activities, such as lipase and production of antibiotics [15, 29]. In the laboratory, primary form cells give rise to secondary form cells after long-term cultivation in liquid media. However, it remains unclear if or when phenotypic variation occurs within the natural life cycle. To accurately determine the production potential of activities in *X. bovienii* strains, we generated secondary form bacterial isolates from primary form isolates and used both for phenotypic testing (Table 2).

**Table 2:**
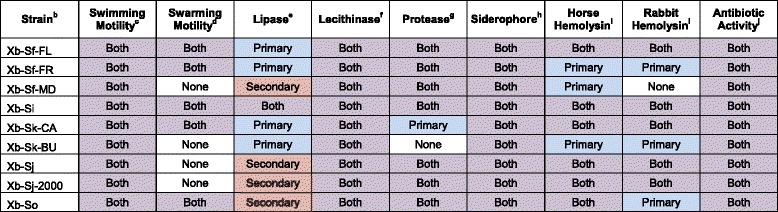
Phenotypes of *X. bovienii* bacterial strains^a^

^a^Phenotypic testing of primary and secondary form *X. bovienii* bacterial strains. Each result is given as the form that produces that activity: Primary – activity in primary form only (blue), Secondary – activity in secondary form only (pink), Both – activity in primary and secondary forms (purple), or None – no activity in either form (no fill)

^b^The bacterial strain tested

^c^Swimming motility detected as motility through soft agar (0.5 % LB agar)

^d^Swarming motility detected as motility on top of semi-solid agar (0.7 % LB agar)

^e^Lipase activity as detected on Tween 20 agar

^f^Lecithinase activity as detected on egg yolk agar

^g^Protease activity as detected on milk agar

^h^Siderophore activity as detected activity on CAS plates

^i^Hemolysin activity as detected on horse blood agar or rabbit blood agar;

^j^Antibiotic activity as detected by antibiotic overlay assays

Almost all tested *X. bovienii* strains have swimming motility, lipase, lecithinase, protease, siderophore, hemolysin, and antibiotic activities in at least one form, except that Xb-Sk-Bu lacks protease activity, and Xb-Sp lacks hemolysin activity (Table 2). Several strains (i.e. Xb-Sf-MD, Xb-Sk-BU, Xb-Sj, Xb-Sj-2000, and Xb-Sp) lack swarming motility (Table 2). Some activities, such as lecithinase and siderophore activity are not affected by phenotypic variation in any of the *X. bovienii* strains. Other activities, such as lipase and hemolysin production, differ between the two forms, and which form produces the activity was inconsistent among the strains. These differences could be due to variations in coding potential among the strains or differences in regulatory control of the genes encoding the activities tested.

To assess if observed activity differences are due to variation in the coding potential, we examined the genomes of the tested *X. bovienii* strains for relevant genes (Additional file 3), such as those encoding hemolysin (*xhlA*, *xhlA2,* and *xaxAB*), lipase (*xlpA*) [17], lecithinase (*estA*) [17], protease (*prtA*) [48], and motility factors (e.g. operons encoding flagellar structural genes and motility regulators) [56]. All of the strains, including those that lack detectable levels of the associated activity, encode intact homologs of these genes (Additional file 3). This indicates that diversity in activity phenotypes is not due to variation in coding potential for structural genes, and we therefore turned our attention to possible differences in regulatory pathways.

Among *Xenorhabdus* species, gene regulation pathways of *X. nematophila* are the best characterized. In this species, the leucine-responsive regulatory protein (Lrp) [57], two-component signal transduction systems CpxRA [6] and OmpR-EnvZ [50], LysR like homolog A (LrhA) [44], flagellar transcriptional regulators (FlhDC) [49, 53, 54], and nematode intestinal localization gene repressor (NilR) [17] regulatory proteins have been implicated in controlling the expression of the phenotypic activities listed in Table 2. Each *X. bovienii* strain genomes contained homologs predicted to encode all of these regulators except NilR (Additional file 4). This is consistent with the fact that in *X. nematophila* NilR functions synergistically to negatively regulate the *nilA, B,* and *C* genes, nematode-host range specificity determinants that are not present in the *X. bovienii* genomes [17, 31, 58].

LysR type transcriptional regulators, of which LrhA is a member, are widely distributed among bacteria, and can respond to specific signals to regulate narrow and broad regulons, which can include genes involved in virulence, metabolism, and other behaviors [59]. We assessed putative LysR type regulators encoded by *X. bovienii* and identified 19 *lysR-*type genes, which are present within all the sequenced *X. bovienii* strains and seven with homologs in one or more strains (Additional file 5). While our inability to identify certain homologs may be due to their absence in draft assemblies, we did identify a *lysR-*type gene within several draft genomes (e.g. XBFFL1v2_2160021) that is absent in the complete Xb-Sj genome. In all of the *X. bovienii* strains examined in which this gene is present, it co-occurs with genes encoding a putative aspartate racemase and a putative glutamate symporter. This analysis indicates that distinctive LysR-type transcription factor regulation of specific metabolic pathways may exist in some strains but not others.

An additional class of regulators common in bacteria is the two-component regulatory systems (TCSs), such as CpxRA and EnvZ/OmpR as mentioned above. These systems transduce specific signals, such as those indicative of a host environment, to various output responses, frequently a change in transcription. In canonical TCSs, the histidine kinase (HK) protein recognizes a stimulus, such as an antimicrobial peptide or quorum sensing molecule, and transmits this signal to the response regulator (RR), which directly or indirectly influences transcription [60]. These transcriptional changes can affect bacterial phenotypic variation [61] and production of virulence factors [62, 63] and degradative enzymes, such as lipase [64]. We assessed the distribution of TCSs among the *X. bovienii* strains, and identified 23 TCSs and 2 orphan RRs that are present in all the strains, and 1 orphan HK and 4 orphan RRs that are within some but not all the sequenced *X. bovienii* strains (Additional file 6). This suggests that differences in TCS-dependent regulation occur among *X. bovienii* strains and could impact their bioactivity.

Our analysis indicates broad conservation of coding potential for transcriptional regulators, and the limited observed variation in the presence or absence of encoded regulators is unlikely to explain the breadth of observed phenotypic differences. Therefore, phenotypic differences among strains may be due to variation in the expression or modulation of regulatory factors. The transcription factor Lrp is of particular interest in this regard as there is an established link between Lrp-dependent regulation and phenotypic variation [57]. However, a detailed understanding of the basis of the observed phenotype differences among the tested *X. bovienii* strains awaits further experimentation examining their individual regulatory hierarchies. The identification of distinct signals to which regulators respond, as well as variations in the constituents of their regulons likely will provide further insights into niche diversification among these bacteria.

### Toxins encoded by *X. bovienii* strains

Bacteria encode a wide range of toxins that are exported by various secretions systems [65]. In symbioses, these toxins can be involved in defensive mutualism (i.e. protection against predators, pathogens, or competitors) or in pathogenesis. For example, lysogenic-phage-encoded toxins expressed by the bacterial secondary symbionts of aphids protect the aphid host from parasitism by parasitoid wasps [66], while lysogenic-phage-encoded Cholera toxin [67] and Shiga toxin [68] are produced during human infection by *Vibrio cholerae* and *Escherichia coli* respectively. In the case of *X. bovienii* bacterial strains, bacterially derived toxins could play a defensive role in protecting the insect cadaver from predators and competitors or could aid in killing the insect host.

**Fig. 3:**
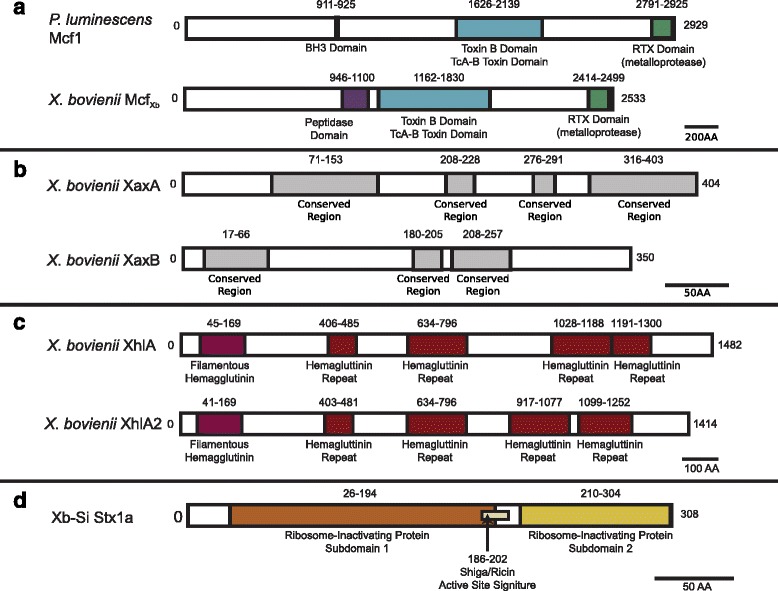
Annotated toxin genes in *X. bovienii* genomes. Schematics represent the domains identified in the proteins of toxin genes: Mcf_Xb_(**a**), hemolysins XaxAB (**b**) and XhlA homologs (**c**), and Shiga toxin Stx1a (**d**). Name of the bacterium encoding each protein and the protein name are listed to the left of each schematic. Colored boxes represent different protein domains or conserved regions, and similar domains are colored the same. The predicted type of domain is listed below, and the amino acids spanning each domain are labeled above. Scale bars represent the amino acid number per distance for each panel

Genome analyses indicated that all of the sequenced *X. bovienii* strains contain genes with homology to makes caterpillars floppy toxin 1 (*mcf1*), metalloprotease MARTX toxin (*MARTX*), zinc alkaline metalloprotease similar to RTX toxins (*prtA*), and hemolysins *Xenorhabdus* alpha-xenorhabdolysin (*xaxAB*) and one or two homologs of *Xenorhabus* hemolysin (*xhlA* and *xhlA2*) (Additional file 7). Homologs of *mcf* have been identified in diverse organisms, including other species of *Xenorhabdus* [39], *Photorhabdus* bacterial symbionts of *Heterorhabditis* nematodes [69, 70] and *Epichloë* fungal symbionts of grasses [71] In the *Photorhabdus – Heterorhabditis* symbiosis, Mcf contributes to virulence towards the insect host and causes the insect to lose body turgor [69, 72]. A common feature of the Mcf toxin homologs is a central TcdA/B translocating domain, but other regions of each Mcf protein contain distinct domains depending on the homolog. The finished *X. bovienii* genome encodes a single homolog [15, 31, 39] that also is present in each *X. bovienii* strain genome analyzed here. Like other *mcf* homologs, these *X. bovienii* homologs encode the conserved TcdA/B like domain as well as a C-terminal RTX toxin-like domain predicted to be involved in export (Fig. 3a) [69]. Additionally, the *X. bovienii* Mcf proteins analyzed here have a putative C58 peptidase domain similar to those in RTX toxins that activate toxins via cleavage (Fig. 3a) [73, 74]. Because of differences between the *X. bovienii* Mcf homologs and the experimentally verified *Photorhabdus* Mcf protein, we refer to the *X. bovienii* homologs as Mcf_Xb_ (Fig. 3a).

Two types of metalloprotease toxins were observed in all of the *X. bovienii* genomes we analyzed. One is homologous to and has the same domain structure as previously identified PrtA proteins from nematode-associated bacterial symbionts *P. luminescens* [48] and *X. nematophila* [75]. The PrtA proteins from *P. luminescens* and *X. nematophila* cleave insect hemolymph proteins and are most likely involved in immunosuppression [76]. The other metalloprotease is a MARTX toxin that has been previously analyzed from the finished *X. bovienii* genome [77, 78]. MARTX metalloprotease toxin proteins, are a family of large RTX toxins containing >40 repeats [79]. Many MARTX proteins are virulence factors, including the cytotoxic RtxA from *Vibrio vulnificus* [80], which is the closest homolog by BLASTp to *X. bovienii* MARTX proteins (named MARTX_Xb_ by convention) [48, 75, 76].

All *X. bovienii* strains analyzed here encode single copies each of *Xenorhabdus* alpha-xenorhabdolysin A and B (XaxA and XaxB), proteins homologous to the XaxAB binary toxin hemolysins of *X. nematophila* [24, 81] and *P. luminescens* [82]. These multiple variants of XaxA and XaxB proteins were used to assess regions of conservation and divergence. We found that each protein had regions (four and three for XaxA and XaxB respectively) with 100 % identity across all homologs (Fig. 3b), while the rest of the regions within the proteins had higher amino acid diversity (Additional file 8D, E). Similar regions of high and low amino acid diversity were present when the XaxAB proteins from *X. nematophila* and *P. luminescens* were included in the analysis (data not shown). These highly conserved regions may be important for XaxAB function, an idea that awaits experimental investigation.

In addition to the XaxAB hemolysin, all analyzed *X. bovienii* strains contain one or two homologs of the *X. nematophila* hemolysin XhlA [25] (Additional file 7). One homolog (XhlA) is present in all of the *X. bovienii* strains, while the other (XhlA2) is present only within a subset of strains (i.e. Xb-Sf-FL, Xb-Sf-FR, Xb-Sf-MD, Xb-Sp and Xb-Si). Both the XhlA and XhlA2 proteins have a similar size (1400 amino acids) (Additional file 8 F) and similar filamentous hemolysin and hemagglutinin repeat domain structures (Fig. 3c).

**Fig. 4:**
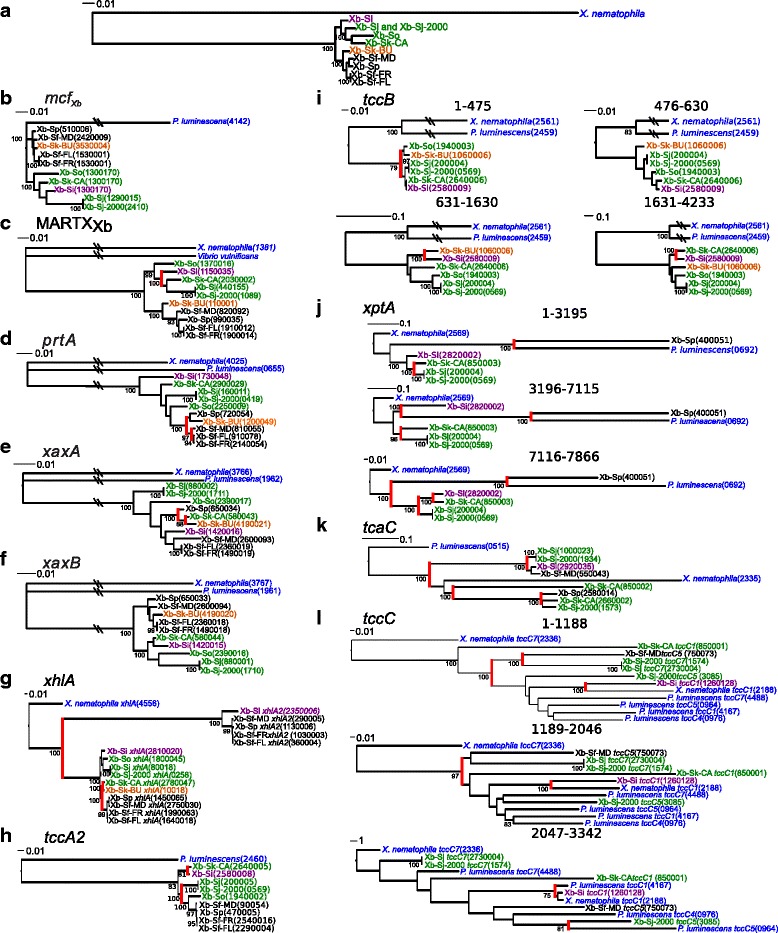
Phylogenies of partitioned nucleic acid sequence. The nucleic acid sequences of genes were analyzed for recombination, and for each piece, phylogenies were built. The gene phylogenies were compared to the whole genome phylogeny of the bacterial strains (**a**) [5]. Shown above are the trees for *X. bovienii* genes *mcf*_*Xb*_ (**b**), *MARTX*_*Xb*_ (**c**), *prtA* (**d**), *xaxA* (**e**), *xaxB* (**f**), *xhlA* (**g**), *tccA2* (**h**), *tccB2* (**i**), *xptA2* (**j**), *tcaC* (**k**), and *tccC* (**l**). Values indicate bootstrap values. The leaves are color-coded by clade determination from the core *X. bovienii* phylogeny, and red highlighting indicates strongly supported branches in the gene tree that do not match the previously reported bacterial phylogeny [5]

To assess if divergence occurs in the toxins discussed above, we assessed each toxin family for amino acid divergence (Additional file 8), recombination, and phylogenetic history (Fig. 4). No intragenic recombination was detected in the nucleotide sequences of any of the toxin families, as analyzed using the break-point analysis method implemented in TOPALi v2 [69]. Also, amino acid sequence divergence was spread evenly throughout each protein, suggesting that each protein region experiences the same level of selection (Additional file 8A-D).

Maximum likelihood nucleotide phylogenetic trees were constructed using full-length sequences from each toxin family, branching orders were compared to the previously published core *X. bovienii* genome phylogeny (Fig. 4a) [5]. The phylogenies for Mcf-family toxin A, *xaxB,* and *xhlA2* matched the previously published core genome phylogeny for these strains at all strongly supported nodes (Fig. 4bf, g), indicating that these genes are orthologs unaffected by horizontal gene transfer (HGT) and likely have conserved functions. Further analyses of Mcf-family toxin A, *xaxB,* and *xhlA2* using PAML v4 [83] revealed that all branches of each phylogeny had dN/dS < 0.30 consistent with negative selection and functional conservation within each class of toxin. Phylogenetic analysis of *MARTX*_*Xb*_*, prtA, xaxA,* and *xhlA* showed that not all strongly supported splits were consistent with the core genome phylogeny, suggesting that HGT had occurred and members of these gene sets are xenologs (Fig. 4 c–e, g). This is consistent with previous findings that toxin genes, such as *xaxAB* are located in regions of genome plasticity (i.e. regions of recombination) in *Xenorhabdus* spp. and are likely transferring between bacteria frequently [39, 84]. Other events that might have caused divergence from the core phylogeny are duplication with subsequent loss of the gene and different rates of evolution, although these are less parsimonious explanations. All of these xenolog sets share the same genomic context, indicating xenologous replacement, and perhaps suggesting functional conservation. Phylogenetic analysis of *xhlA* and *xhlA2* indicate that these genes likely arose from a gene duplication event and are paralogs of one another (Fig. 4g). This supports the XhlA and XhlA2 proteins potentially having divergent functions. Taken together, our divergence, recombination, and phylogenetic analyses have revealed that all of the toxin classes we analyzed likely have conserved function in the encoding *X. bovienii* strains, with the exception being in the hemolytic enzymes encoded by *xhlA* and *xhlA2.*

In addition to the shared toxins that are described above, the symbiont from *S. intermedium,* Xb-Si also encodes a putative Shiga toxin chain A (Additional file 7). This gene was previously identified through analyses of proteins unique to each *X. bovienii* strain [5]. The top matches for this protein from BLASTp indicate that it is most similar (e-value = 1.0E-37) to Shiga toxin 1 variant A from *Escherichia coli* (Additional file 9), and analysis of its domain structure revealed two ribosome-inactivating protein subdomains as well as a signature Shiga-type active site (Fig. 3d) [85]. Examination of the surrounding proteins and total genome content did not reveal an obvious Shiga toxin B chain homolog, which is responsible for targeting the toxin complex to the correct mammalian cell type by binding a cell-surface ganglioside [86]. As *X. bovienii* is not known to colonize a mammalian or other vertebrate host, we hypothesize that it instead encodes an invertebrate-specific targeting protein partner for the Shiga toxin variant A homolog. The genes surrounding the Xb-Si Shiga toxin are of unknown function or are phage associated (i.e. holin and transposase). This suggests that the toxin may have been transferred via a phage, similar to Shiga toxin producing *E. coli* or *Vibrio cholerae* [87]. Analysis of the predicted holin and transposase did not conclusively identify the type of phage from which they were derived.

### Insecticidal toxin complexes are diverse among *X. bovienii* bacterial strains

In addition to the shared annotated toxins described in the previous section, *Xenorhabdus* and *Photorhabdus* bacteria encode insecticidal toxin complex (Tc) proteins [88, 89]. Tc toxins are large molecular weight toxins comprised of three protein subunits: A, B, and C. For each subunit, there are multiple genes that can encode a similar function (e.g. *xptA2* and *tccA2* both encode A subunit proteins) [88, 89]. Recent literature suggests that all three protein subunits function together for secretion from the cell, binding of the target membrane, and translocation into the cell to deliver the C-terminal end of the C subunit, which is the functional toxin [90–92]. The B subunit has also been proposed to function in linking the A and C subunits. The A subunit mediates host cell targeting and specificity of the toxin complex through membrane receptor binding of insect intestinal cells [89, 91, 93]. Additionally, some A subunit proteins (XptA1 and XptA2) have independent oral toxicity against insects [89, 93].

The finished genome of *X. bovienii* (Xb-Sj-2000) encodes three intact A subunits, two intact B subunits, and two intact C subunits [31]. To determine if genes encoding Tc toxins have diversified within different strains of *X. bovienii*, we identified homologs in the 9 draft genomes using a keyword search of the genome annotations and a BLASTp search of the Tc toxin proteins previously identified in the Xb-Sj-2000, *X. nematophila*, and *P. luminescens* genomes (Additional file 10). We identified 118 potential A, B, and C subunit genes within the 10 *X. bovienii* bacterial genomes with 4–20 genes in each genome, but further analysis revealed that many of the genes are not predicted to encode full length protein subunits (i.e. were truncated versions of the proteins identified in other *X. bovienii* strains) (Additional file 10). The non-full-length genes are located in the middle of contigs that are otherwise syntenous with Xb-Sj-2000, indicating that truncation likely is not due to incorrect assembly. Additionally, Xb-Sj lacked one of the seven Tc toxin genes that were found in Xb-Sj-2000, supporting that the majority of differences are not due to misassembly. All genomes had fragmented open reading frames corresponding to each subunit type, but it is unclear if these fragments would be able to function as a complete protein when combined. When considering only full length coding regions, all of the sequenced *X. bovienii* strains encoded at least one of three potential A subunits (XptA2, TccA2, TccB2), five strains encoded an intact B subunit (TcaC), and six strains encoded an intact C subunit (TccC) (Additional file 10). Only four (Xb-Sf-MD, Xb-Sk-CA, Xb-Sj, Xb-Si) of the nine draft genomes encode at least one intact protein of each subunit type.

To assess the variability among intact Tc protein sequences, we measured amino acid divergence between these proteins (Additional file 8) and constructed nucleotide and amino acid sequence phylogenies (Additional file 11). Separate analysis of the three A subunit types (i.e. XptA2, TccA2, TccB2) revealed that substitutions in amino acid sequences among the homologs are distributed evenly throughout the amino acid sequence (i.e. no regions of the protein showed greater number of substitutions than others) (Additional file 8G-I). There are two areas of XptA2 amino acid sequence that are somewhat less conserved than the rest of the protein, but the majority of this diversity is driven by Xb-Sp (Additional file 8G). For B subunit proteins, amino acid substitutions were distributed along the length of the protein (Additional file 8J). Phylogenetic analysis showed that these proteins clustered into two clades (Additional file 11D), indicating some combination of horizontal transfer and/or gene duplication events in the evolutionary history of these genes and potential functional divergence. Consistent with previous reports, *tccC* genes had greater sequence divergence near the C-terminal end of the protein (Additional file 8K) [31]. This portion of the protein is the functional domain. BLASTp analysis found that each TccC protein showed the highest similarity to one of four subtypes (TccC1, TccC4, TccC5, TccC7) (Additional file 10). However, different portions of each TccC protein matched different subtypes, including some multiple matches (although with different strength). These data indicate that overall amino acid similarity does not accurately annotate the correct subtype for an individual TccC protein.

To further characterize protein divergence, we performed phylogenetic and dN/dS analyses of Tc toxin amino acid and nucleotide sequences. Using TOPALi analysis, there are 2 recombination points leading to 3 distinct recombination blocks in *xptA2*. In bacterial pathogens, recombination leads to shuffling of virulence genes and can confer new ecological niches on recipient bacteria [94, 95]. Phylogenetic analysis of the *xptA2* nucleotide sequences encoded by each recombination block from TOPALi analysis identified that the phylogenetic tree for each portion was distinct and each phylogeny had some branching patterns not congruent with the *X. bovienii* core genome phylogenetic tree, consistent with recombination [5] (Fig. 4j). The *xptA2* genes in Xb-Sp and Xb-Sk-CA shared the same genomic context, as did those in Xb-Sj-2000, Xb-Sj, and Xb-Si. In contrast, the analyzed full-length *X. bovienii tccA2* and *tccB2* (A subunit-encoding) genes have not undergone recombination according to TOPALi, but have potentially undergone HGT, as not all strongly supported nodes were congruent with the *X. bovienii* core genome phylogeny (Fig. 4h, i). However, The *tccA2* and *tccB2* xenologs have the same genomic context and therefore may have conserved functions.

Phylogenetic analysis of the Tc toxin B subunit proteins showed that proteins clustered into two clades (Additional file 11D), indicating potential horizontal transfer and/or gene duplication. We did not identify recombination among B subunit genes using TOPALi [5] (Fig. 4k). However, all *tcaC* (B subunit-encoding) genes except that of Xb-Sp have the same genomic context, and therefore may have conserved functions.

Analysis of the Tc toxin C subunit *tccC* genes indicated that a recombination breakpoint occurred 1188 bp from the 3’-end of these genes, consistent with increased sequence diversity within this region, as well at 2047 from the 3’-end of these genes [31] (Fig. 4l). Phylogenetic analyses of nucleotide sequences from each of these recombination blocks had different topologies, consistent with recombination. Parallel amino acid phylogenies lacked sufficient phylogenetic to display such incongruities (Additional file 4E-G). Additionally, not all strongly supported splits were consistent with the core genome, suggesting potential horizontal transfer of whole genes. Xb-Sj-2000 has two copies of the *tccC* genes, suggesting potentially divergent functions between them. The *tccC* genes from Xb-Sj, Xb-Sj-2000, and Xb-Sk-CA have the same genomic context, unlike those from Xb-Sf-FR, Xb-Sf-MD, and Xb-Si, which each has a distinct genomic context. Together, these data indicate that *X. bovienii tccC* genes have complicated recent evolutionary histories and that their functions likely vary among *X. bovienii* strains.

There is considerable divergence among the previously characterized Tc toxin proteins and those identified in *X. bovienii*, indicating that *X. bovienii* toxin proteins may target different insects or have different effects on target cells and molecules. As insecticidal toxins, these proteins may function in the symbiosis to aid in killing the insect host or to protect the insect cadaver against insect scavengers. Indeed, one study has shown that Tc toxins are dispensable for virulence in *Xenorhabdus* strains, at least in particular insect hosts, suggesting they may play other roles in the life cycle in addition to or instead of insecticidal activity [39]. Therefore, variation in the Tc toxins among *X. bovienii* strains likely reflects differences in the insect hosts or scavengers encountered during their lifecycle.

### Non-ribosomal peptide synthetase and polyketide synthetase cluster variation

A class of molecules likely involved in symbiotic interactions is produced by non-ribosomal peptide synthetase (NRPS) and polyketide synthetase (PKS) systems: multi-gene, modular enzymes that synthesize small molecules with a variety of biological functions. Prior studies have reported extensive diversity in NRPS and PKS clusters among entomopathogenic nematode symbiont species, such as *X. nematophila* and *P. luminescens* [18, 31]. Among *X. bovienii* bacterial strains, variation in NRPS and PKS coding potential also occurs (Table 3). A total of 29 distinct NRPS and PKS clusters were identified among all the analyzed strains combined. Of these clusters, six were identified in all of the strains (conserved) and seven were found within only one strain (unique). The remaining sixteen clusters were found in a subset of some, but not all, strains (shared). While some differences in NRPS and PKS clusters could be due to genome misassembly, as these are repetitive sequences, the two like genomes (Xb-Sj and Xb-Sj-2000) only differ by one cluster. This suggests that the majority of clusters did assemble correctly.

**Table 3:**
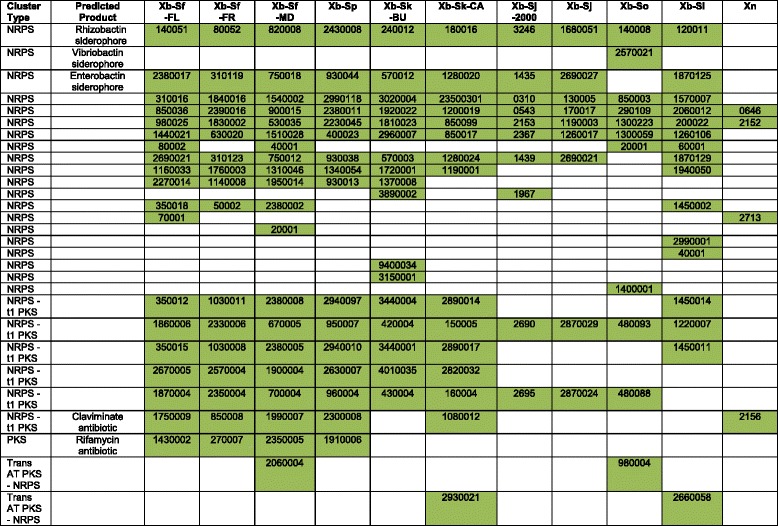
NRPS an PKS clusters^a^

^a^Distribution of NRPS and PKS clusters among the X. bovienii genomes listed by the biosynthesis gene of the cluster that js numerically first in the genome annotation. The number designation for each gene is given without the prefixes: Xb-Sf-FL (XBFFL1), Xb-Sf-FR (XBFFR1), Xb-Sf-MD (XBFMD1), Xb-Sp (XBP1), Xb-Sk-BU (XBKB1), Xb-Sk-CA (XBKQ1), Xb-Sj-2000 (XBJ1), Xb-Sj (XBJ2), Xb-So (XBO1), Xb-Si (XBI1), and Xn (XNC1)

^b^Types of clusters: NRPS (non-ribosomal peptide synthetase), PKS (polyketide synthetase), T1 PKS (type 1 PKS), or Trans AT PKS (trans-acyltransferase PKS)

^c^Predicted product from the NRPS and PKS clusters, if known

One of the conserved clusters, one of the unique clusters, and four shared clusters have known siderophore (i.e. metal scavenging compounds) or antibiotic products (Table 3). The remaining clusters (five conserved, six unique, and 12 shared clusters) do not have a known product. When compared to the published genomes of *X. nematophila* and *P. luminescens* [31, 32] we found that of the 29 identified clusters, six (two conserved, one unique, and three shared clusters) were also shared by *X. nematophila* and none were shared by *P. luminescens* (Table 3). This indicates that entomopathogenic *Xenorhabdus* and *Photorhabdus* symbiont species all encode many NRPS and PKS clusters and likely produce many different secondary metabolite products.

The function of *Xenorhabdus* NRPS and PKS products are diverse, and include anti-microbial [11, 18, 96–98], anti-predation [8, 9, 99], immunosuppressive [100], hemolytic [98], and metal acquisition [101] activities. Several of these functions have the potential to provide symbiotic benefits. Products that provide anti-microbial or anti-predation activity could play a defensive role within the symbiosis by protecting the insect cadaver, and therefore the developing nematodes and bacteria, from invasion by pathogens, competitors, or predators. Additionally, products that provide immunosuppressive or hemolytic activity could aid in the killing of the insect host, thereby providing nutrition to the nematodes and bacteria. Although it is possible that the predicted NRPS and PKS cluster products provide these benefits, defining the activities and role of the molecules awaits further experimental testing and functional characterization.

## Conclusions

Overall, the presented analyses highlight that fundamental processes underlying symbiotic interactions in *X. bovienii* (e.g. secretion systems and degradative activities) are largely conserved between strains. However, phenotypic testing indicates that despite conserved coding potential, strain-level differences in symbiosis factor expression occurs (Table 2). Further, we observed notable variation in certain classes of genes, such as those encoding NRPS and PKS clusters and Tc toxins (Table 3, Additional file 10). This variation may contribute to previously identified differences in symbiotic fitness [5], but confirmation of this possibility awaits further experimental assessment.

By standard molecular metrics (e.g. average nucleotide identity and 16S rRNA analyses) the *X. bovienii* strains analyzed here previously were identified as members of the same species [5, 30]. Our data demonstrate that within the *X. bovienii* species, there is diversity in the coding potential of bioactive molecules (Table 3, Additional file 10) and the regulation of some conserved products [5] (Table 2). These data in combination with the finding that these strains differ in symbiotic fitness [5] suggest that using ecotype definitions, rather than bacterial species or strains, might be more useful in categorizing diversity among bacterial symbionts and their potential impact on host fitness. Bacterial ecotypes are defined as evolutionarily and ecologically distinct groups [102] and within symbiosis, this could be defined as groups that produce particular molecules to interact with specific hosts. The use of bacterial ecotype definitions would allow for detection and discussion of the nuanced differences among symbiont groups.

Variation in the ability of the bacterial strains to engage in symbiosis with the nematode likely results from coevolution, and therefore co-adaptation, between nematode host species and bacterial symbiont strains [4, 5]. It is also possible that differences in the ability of symbionts to engage in symbiosis could be due to differential gene loss or access to gene pools, not necessarily dependent on coevolution. Activities predicted to diversify in this way are those that benefit the nematode host, such as those involved in nutrient acquisition. Our data indicate that while many of the nutritional factors do not differ among strains, their regulation does (e.g. lipase). This demonstrates that while overall nutritional requirements may not vastly differ between nematode hosts, the timing of expression of metabolic pathways varies and may be important for optimal symbiotic benefits. We did not detect any phenotypic pattern that was consistent with the published nematode or bacterial phylogenies [5] (data not shown), suggesting that expression of the measured activities is dependent on variables other than or in addition to the nematode host identity, such as the identity of the insect host. Likewise, bacterial factors that contribute to virulence towards the insect host or to defense against predators likely vary based on selective pressures of environmental differences encountered by the bacterial strains, such as the insect host species, endogenous bacterial competitors within these insects, or the predators, competitors, and pathogens naturally encountered. Our genomic analyses demonstrate diversity in many of these types of compounds (e.g. NRPS derived molecules, Tc toxins, and hemolysins), suggesting that the nematode – bacterial pairs encounter different insect hosts and competitors, as some of these have been demonstrated to function differently against divergent insect hosts [93]. However, many of the large molecular weight toxins (e.g. Mcf_Xb_, MARTX_Xb_ toxin, and XaxAB) are conserved in all *X. bovienii* strains, indicating that these toxins may not be specific to particular insect host-ranges and instead functional against many insect hosts. Unfortunately, little is known about the identity of *X. bovienii* insect hosts, predators, competitors or pathogens that occur in natural conditions, precluding concrete conclusions correlating the diversity of gene products to naturally encountered organisms. However, our comparative genomics and phenotypic analyses do support that diversity in symbiotic functions of the *X. bovienii* bacterial strains differ due to the diversity of effectors and their regulation rather than the utilization of different mechanisms for interacting with hosts.

In addition to providing insight into the diversity of potential symbiotic functions, the data presented here highlight that strain variability is an important consideration when exploiting *Xenorhabdus* bacteria for discovery of compounds for application purposes. For the discovery of novel antibiotics or other NRPS- and PKS-derived compounds from *Xenorhabdus* spp., it will be useful to assess multiple bacterial strains, as we observed large strain-level diversity in coding potential for these systems. This is also the case when assessing Tc toxin clusters. However, for the application of many other large molecular weight toxins (e.g. XaxAB, Mcf_Xb_, MARTX_Xb_), the activities determined from a single bacterial strain likely will be similar among the members of that species but divergent from the toxins identified in different *Xenorhabdus* species.

In summary, the comparative genomic analysis presented here provides an assessment of *X. bovienii* bacterial strain variation in factors that could be involved in symbiotic interactions and may be utilized for applications. This analysis provides a foundation for understanding how bacterial strain variability affects symbiosis and for the discovery of novel compounds within *Xenorhabdus* spp.

## Methods

### Genome features

Genomes were submitted to MaGe [103, 104] for annotation and analysis. The genomes were analyzed for size, GC content, number of coding sequences, and the percentage of the genome covered by coding content. The genomes were assessed for synteny using MAUVE 2.3.1 [105] relative to the finished genome of *X. bovienii* (Table 1) [32]. This analysis was done excluding contigs <1000 bp.

### Plasmid detection

No plasmids were detected in any of the *X. bovienii* strains. The experimental methods utilized were, plasmid extraction kit Zippy Plasmid Miniprep Kit (Zymo, Irvine, CA), plasmid boil prep [106], and Wizard Genomic DNA Purification Kit (Promega, Madison, WI). After extraction, DNA was run on a 1 % agarose gel and observed for bands. No bands corresponding to potential plasmid were observed. Additionally, within DNA sequences, no genes corresponding to know plasmid genes (e.g. origin of replication) were identified using BLASTp. This BLAST analyses, and all others performed in this study were done using default parameters, and a significant match was considered 75 % coverage with an e-value <1E-4.

### Global metabolism analysis

The metabolic pathways of each strain were determined using two methods. The MaGe platform [103, 104] was utilized to interpret the percentage of each pathway that was intact, and this platform uses KEGG and EC annotations. Additionally, pathways were analyzed through mapping to MetaCyc [107], which is shown as the number of pathways present for each class. Genomes were compared for metabolic coding potential by identifying if pathways were divergent among the strains in both analyses. Incompleteness of a pathway was confirmed through BLASTp analysis using missing pathway enzymes.

### Secretion systems

Secretion systems were identified through keyword searching the MaGe annotations for secretion system components (Additional file 1). For a secretion system to called intact, it must have had all known necessary secretion system genes [35, 36, 40, 42, 45–47, 108]. The presence or absence of other secretion systems were assessed through comparing known necessary secretion system components to the genomes using BLASTp [109].

### NRPS and PKS clusters

NRPS and PKS clusters were identified by analyzing each genome using antiSMASH 3.0 [110]. Clusters were confirmed as intact by assessing that each cluster was in the middle of a contig and the genes encoding the cluster were surrounded by genes annotated as other functions. Exclusion of clusters on the edge of contigs resulted in the exclusion of 23 clusters (1–3 per genome). The NRPS and PKS clusters were compared to determine their distribution among *X. bovienii* genomes by assessing the cluster proteins for local synteny within the genomes, using MaGe and MAUVE 2.3.1 alignments. The presence or absence of the *X. bovienii* NRPS and PKS clusters in *X. nematophila* and *P. luminescens* was determined by searching for the gene clusters in the finished genomes using MaGE synteny mapping.

### Toxins

Genes for putative toxin proteins were revealed by searching for genes annotated as toxins by the MaGe platform. Annotations were further supported by BLASTp, Interpro 51.0 [111], and Swiss Prot [103, 104] analyses. Toxin domains were analyzed using Interpro, and in the case of Mcf1, comparison to known protein domains in homologs. Assessment of toxin subtypes (i.e. Shiga toxin and Tc toxin) was performed based on BLASTp results [109]. For assessing similarities among *X. bovienii*, amino acid sequences were aligned using MUSCLE 3.7 [112, 113], and protein distances were calculated using Phylip 3.695 with the Jones-Taylor-Thornton model in Protdist [114]. Protein distances are given in point accepted mutation (PAM), representing the number of point mutation events in 100 amino acids. Protein trees were built using Maximum Likelihood and bootstrapped in Phylip [114], and trees were visualized in iTol [115]. For visualization of amino acid differences along the length of the proteins, the alignment was visualized in MegAlign Pro from DNASTAR 11.0 (www.dnastar.com). Regions of dissimilarity were considered when at least four amino acids in a row were not conserved among the homologs.

### Testing of recombination, horizontal gene transfer, and selection

Nucleotide sequences were aligned in MEGA v6.0 [116]. Analyses of nucleotide sequences was done in TOPALi v2 [117]. Assessment of recombination was done in TOPALi using DSS and the sequence alignment was divided up into portions with a similar evolutionary history. Horizontal gene transfer was identified by comparing gene phylogenies to the bacterial whole genome phylogeny [5]. Phylogenetic trees were built in TOPALi from the full length or sub-sections of the nucleotide sequences as determined by DSS. Bootstrapping was done using the maximum likelihood GTR substitution model in Topali. Bootstrap values were used to determine strongly supported splits (>75 %). Selection was determined using PAML in TOPALi to calculate dN/dS ratios [118].

### Phenotypic testing

Stable secondary form (variant 2) bacterial strains were isolated from stable primary form (variant 1) bacteria through repeated passage. Briefly, bacterial strains were grown at 30 °C in lysogeny broth with aeration in the dark. Bacterial strains were grown approximately 24 h and sub-cultured into fresh media. Sub-culturing occurred for a period of 2–4 weeks, until bacteria spread on NBTA agar [119] no longer bound bromothymol bule dye and was red in color with repeated restreaking. Phenotypic tests were performed using previously published assays for swimming [120] and swarming [54] motility on LB agar, lipase on tween 20 agar [121], lecithinase on egg yolk agar [122], protease on milk agar [122], siderophore on CAS agar [52], and hemolysin on horse and rabbit blood agar plates [123]. For all assays, 5 μL of overnight bacterial culture was spotted onto the agar plate and dried, and the plates were incubated at 30 °C in the dark for 48 h prior to reading. Antibiotic activity was determined through overlay assays using *Escherichia coli*, *Micrococcus luteus,* and *Bacillus subtilis* as the overlayed test strains [124]. For antibiotic activity, 5 μL of overnight *X. bovienii* bacterial culture was spotted onto LB agar plates supplemented with pyruvate and dried, and the plates were incubated for at 30 °C in the dark for 24 h prior to overlaying with the test bacterial strain. After overlay, the plates were incubated for at 37 °C for 24 h prior to reading. For these experiments, all test strains were inhibited. For all activities, the relative activity was measured through measuring the diameter of the output (e.g. zone of clearing). However, no significant difference was observed between strains with measurable activity. Therefore the activity is reported as yes or no rather than numerically.

### Assessment of *X. bovienii* genes contributing to phenotypic activity

Homologs of genes known to contribute to the identified activity were assessed through BLASTp analysis of the homologs in each *X. bovienii* bacterial strain. NRPS and PKS clusters were determined as described above.

### Availability of data and materials

Genomes have been deposited in GenBank (http://www.ncbi.nlm.nih.gov/genbank/) (Table 1).

## Additional files

Additional file 1: Table S1. Global metabolic analysis of *Xenorhabdus bovienii* strains. Description: Table of all metabolic pathways in *X. bovienii* and the relative completeness of each pathway. (XLSX 76 kb) Additional file 2: Table S2. Secretion system genes. Description: Table of all genes identified encoding the complete secretion systems in *X. bovienii* bacterial strains. (DOC 78 kb) Additional file 3: Table S3. Bacterial genes predicted to encode select symbiotic activities. Description: Table of all genes identified within the bacterial strains that could confer the activities observed in Table 2. (DOC 32 kb) Additional file 4: Table S4. Regulatory proteins found within *X. bovienii* strains. Description: Table of all genes predicted to encode the well-characterized regulatory proteins in *Xenorhabdus* species. (DOC 35 kb) Additional file 5: Table S5.
*Xenorhabdus bovienii* genes predicted to encode LysR family transcription factors. Description: Table of all genes within all *X. bovienii* strains predicted to encode LysR family regulators. (DOC 62 kb) Additional file 6: Table S6.
*Xenorhabdus bovienii* genes predicted to encode two-component regulatory systems. Description: Table of all genes within all *X. bovienii* strains predicted to encode two-component regulators. (DOC 74 kb) Additional file 7: Table S7. Annotated toxin genes. Description: Table of all genes within all *X. bovienii* strains predicted to encode toxin proteins excluding Tc toxin subunits. (DOC 34 kb) Additional file 8: Figure S1. Distribution of amino acid sequence divergence in proteins. Description: Image of the amino acid sequence divergence among *X. bovienii* homologs of Mcf_Xb_, MARTX_Xb_, PrtA, XaxA, XaxB, XhlA/XhlA2, XptA2, TccA2, TccB2, TcaC, and TccC. (PDF 181 kb) Additional file 9: Table S8. Best BLASTp hits of Xb-Si putative Shiga toxin. Description: Table of the top 5 BLASTp hits for the putative Shiga toxin from Xb-Si (XbI1v2_2730004). (DOC 30 kb) Additional file 10: Table S9. Tc subunit genes from *X. bovienii* genomes. Description: Table of all predicted Tc toxin subunit proteins from all *X. bovienii* genomes, including the intact and fragmented open reading frames. (DOC 128 kb) Additional file 11: Figure S2. Phylogenies of TC toxin amino acid sequences. Description: Image of the phylogenies built from amino acid sequences of *X. bovienii* homologs XptA2, TccA2, TccB2, TcaC, and TccC. (PDF 52 kb)
