# Supplementary material for: Comparison of Xenorhabdus bovienii bacterial strain genomes reveals diversity in symbiotic functions
Source: BMC Genomics. 2015 Nov 2;16:889. doi: 10.1186/s12864-015-2000-8 (PMC4630870; doi:10.1186/s12864-015-2000-8)
Supplement: Additional file 2: Table S2. — Secretion system genes. Description: Table of all genes identified encoding the complete secretion systems in X. bovienii bacterial strains. (DOC 78 kb) [file 12864_2015_2000_MOESM2_ESM.doc]

**Additional File 2: Table S2. Secretion system genes.**

| **Secretion System^a^** | **Gene Name^b^** | **Gene Annotation^c^** |
| --- | --- | --- |
| Type II | *ffh* | XBJ1_3270 |
| Type II | *ftsY* | XBJ1_0553 |
| Type II | *lepB* | XBJ1_3149 |
| Type II | *lexA* | XBJ1_3997 |
| Type II | *lspA* | XBJ1_1725 |
| Type II | *secA* | XBJ1_3467 |
| Type II | *secB* | XBJ1_4332 |
| Type II | *secD* | XBJ1_1677 |
| Type II | *secE* | XBJ1_4060 |
| Type II | *secF* | XBJ1_1676 |
| Type II | *secG* | XBJ1_0365 |
| Type II | *secM* | XBJ1_3468 |
| Type II | *secY* | XBJ1_4221 |
| Type II | *sppA* | XBJ1_2447 |
| Type II | *yajC* | XBJ1_1678 |
| Type II | *yidC* | XBJ1_4410 |
| Type VI | *clpV* | XBJ1_0271 |
| Type VI | *icmF* | XBJ1_0275 |
| Type VI | *impA* | XBJ1_0274 |
| Type VI | *impB* | XBJ1_0262 |
| Type VI | *impC* | XBJ1_0263 |
| Type VI | *impG/vasA* | XBJ1_0265 |
| Type VI | *impH/vasB* | XBJ1_0266 |
| Type VI | *impJ/vasE* | XBJ1_0269 |
| Type VI | *impK/vasF* | XBJ1_0270 |
| Type VI | *hcp/tssD* | XBJ1_0261 |
| Type VI | *vasD* | XBJ1_0268 |
| Type VI | *vasI* | XBJ1_0273 |
| Type VI | *vgrG* | XBJ1_0277 |
| Type VI | *vgrG* | XBJ1_0302 |
| Flagellar | *dsbB* | XBJ1_2460 |
| Flagellar | *flgA/flaU* | XBJ1_1949 |
| Flagellar | *flgB/flbA* | XBJ1_1950 |
| Flagellar | *flgC/flaW* | XBJ1_1951 |
| Flagellar | *flgD/claV* | XBJ1_1952 |
| Flagellar | *flgE/flaK* | XBJ1_1953 |
| Flagellar | *flgF/flaX* | XBJ1_1954 |
| Flagellar | *flgG/flaL* | XBJ1_1955 |
| Flagellar | *flgH/flaY* | XBJ1_1957 |
| Flagellar | *flgI/flaM* | XBJ1_1959 |
| Flagellar | *flgJ/flaZ* | XBJ1_1960 |
| Flagellar | *flgK/flaS* | XBJ1_1961 |
| Flagellar | *flgL/flaT* | XBJ1_1962 |
| Flagellar | *flgN* | XBJ1_1947 |
| Flagellar | *flhA/flaH* | XBJ1_1939 |
| Flagellar | *flhB/flaG* | XBJ1_1938 |
| Flagellar | *flhC/flaI* | XBJ1_1918 |
| Flagellar | *flhD/flbB* | XBJ1_1917 |
| Flagellar | *fliA/flaD* | XBJ1_1996 |
| Flagellar | *fliD/flbC* | XBJ1_1994 |
| Flagellar | *fliE/flaN/flaAI* | XBJ1_2703 |
| Flagellar | *fliF/flaBI* | XBJ1_2704 |
| Flagellar | *fliG* | XBJ1_2705 |
| Flagellar | *fliH* | XBJ1_2706 |
| Flagellar | *fliJ/flaO* | XBJ1_2708 |
| Flagellar | *fliK/flaE* | XBJ1_2709 |
| Flagellar | *fliS* | XBJ1_1993 |
| Flagellar | *fliT* | XBJ1_1992 |
| Flagellar | *motA/flaJ* | XBJ1_1919 |
| Flagellar | *motB/flaJ* | XBJ1_1920 |
| Protease Secretion | *arpD* | XBJ1_0489 |
| Protease Secretion | *arpE* | XBJ1_0488 |
| Protease Secretion | *arpF* | XBJ1_0487 |

^a^Secretion system that the gene is part of.

^b^Gene annotation.

^c^Number designation for the gene in the Xb-Sj-2000 genome. All genomes encode these genes, although only Xb-Sj-2000 designations are shown.
