# Supplementary material for: Comparison of Xenorhabdus bovienii bacterial strain genomes reveals diversity in symbiotic functions
Source: BMC Genomics. 2015 Nov 2;16:889. doi: 10.1186/s12864-015-2000-8 (PMC4630870; doi:10.1186/s12864-015-2000-8)
Supplement: Additional file 3: Table S3. — Bacterial genes predicted to encode select symbiotic activities. Description: Table of all genes identified within the bacterial strains that could confer the activities observed in Table 2. (DOC 32 kb) [file 12864_2015_2000_MOESM3_ESM.doc]

**Additional File 3: Table S3. Bacterial genes predicted to encode select symbiotic activities^a^ .**

| **Activity^b^** | **Predicted gene product(s)^c^** | **Predicted gene(s)^d^** | **Citation(s)^e^** |
| --- | --- | --- | --- |
| **Swimming and Swarming motility** | flagellar structural components and regulators | *flgA-M, flhA-D, fliAE-TY, flaA-CENP-R, ompR/envZ* | 42-44, 49, 53, 54 |
| **Lipase** | phospholipase A1 | *xlpA* | 17 |
| **Lecithinase** | esterase | *estA* | 17 |
| **Protease** | alkaline protease | *prtA* | 48, 75 |
| **Siderophore** | small molecule siderophore | *xhbF*, several NRPS/PKS clusters | 51, 52, 101 |
| **Horse and rabbit hemolysin** | secreted hemolysin | *xhlAB*, xaxAB, several NRPS/PKS clusters | 24, 25, 82, 91 |
| **Antibiotic activity** | small molecules | several NRPS/PKS clusters | 11, 18, 89, 96-100 |

^a^List of genes and gene products predicted to encode the activities measured in Table 4.

^b^Activity measured as listed in Table 4.

^c^Gene product that would likely produce the measured activity.

^d^Annotated genes from *X. bovienii* genomes that could produce the measured activity.

^e^Citation for the gene-function connection.
