# Supplementary material for: Comparison of Xenorhabdus bovienii bacterial strain genomes reveals diversity in symbiotic functions
Source: BMC Genomics. 2015 Nov 2;16:889. doi: 10.1186/s12864-015-2000-8 (PMC4630870; doi:10.1186/s12864-015-2000-8)
Supplement: Additional file 4: Table S4. — Regulatory proteins found within X. bovienii strains. Description: Table of all genes predicted to encode the well-characterized regulatory proteins in Xenorhabdus species. (DOC 35 kb) [file 12864_2015_2000_MOESM4_ESM.doc]

**Additional File 4: Table S4. Regulatory proteins found within *X. bovienii*** strains.

| **Gene** | **Xb-Sf-FL (XBFFL1v2_)** | **Xb-Sf-FR (XBFFR1v2_)** | **Xb-Sf-MD (XBFM1**  **v2_)** | **Xb-Si**  **(XBI1v2_)** | **Xb-Sj**  **(XBJ2**  **v2_)** | **Xb-Sj-2000**  **(XBJ1_)** | **Xb-Sk-BU**  **(XBKB1v2_)** | **Xb-Sk-CA**  **(XBKQ1v2_)** | **Xb-So**  **(XBO1**  **v2_)** | **Xb-Sp**  **(XBP1**  **v2_)** |
| --- | --- | --- | --- | --- | --- | --- | --- | --- | --- | --- |
| ***lrp*** | 90006 | 20006 | 2680044 | 2380049 | 430007 | 0890 | 80006 | 2220007 | 600065 | 10006 |
| ***cpxRA*** | 1090026  1090027 | 1130027  1130028 | 1910032  1910033 | 1860003  1860004 | 2390062  2390063 | 4311  4312 | 4140046  4140047 | 2880005  2880006 | 2530021  2530022 | 620048  620049 |
| ***ompR – envZ*** | 2170097  2170107 | 2050016  2050006 | 2050020  2050010 | 2810083  2810073 | 60035  60046 | 0186  0197 | 1240066  NA | 1840014  NA | 2510053  2550020 | 2200028  1450030 |
| ***lrhA*** | 1190023 | 2070078 | 1740082 | 3010081 | 1560016 | 2926 | 440019 | 1850085 | 970022 | 3010058 |
| ***flhDC*** | 2770014  2770015 | 1260014  1260015 | 2140014  2140013 | 2920050  2920049 | 1000010  1000011 | 1917  1918 | 3820015  3820014 | 2240014  2240013 | 480012  480013 | 3080013  3080014 |
| ***nilR*** | NA | NA | NA | NA | NA | NA | NA | NA | NA | NA |

Table of genes annotated as homologs to regulatory proteins in *X. bovienii* genomes as determined by MaGe and listed as the annotated gene. The number designation for each gene(s) is given without the prefixes, which are listed at the top of each column. HK in gene column designates and unknown histidine kinase, while RR designates an unknown response regulator.
