# Supplementary material for: Comparison of Xenorhabdus bovienii bacterial strain genomes reveals diversity in symbiotic functions
Source: BMC Genomics. 2015 Nov 2;16:889. doi: 10.1186/s12864-015-2000-8 (PMC4630870; doi:10.1186/s12864-015-2000-8)
Supplement: Additional file 5: Table S5. — Xenorhabdus bovienii genes predicted to encode LysR family transcription factors. Description: Table of all genes within all X. bovienii strains predicted to encode LysR family regulators. (DOC 62 kb) [file 12864_2015_2000_MOESM5_ESM.doc]

**Additional File 5: Table S5. *Xenorhabdus bovienii* genes predicted to encode LysR family** transcription factors.

| **Gene** | **Xb-Sf-FL (XBFFL1v2_)** | **Xb-Sf-FR (XBFFR1v2_)** | **Xb-Sf-MD (XBFM1v2_)** | **Xb-Si**  **(XBI1v2_)** | **Xb-Sj**  **(XBJ2v2_)** | **Xb-Sj-2000**  **(XBJ1_)** | **Xb-Sk-BU**  **(XBKB1v2_)** | **Xb-Sk-CA**  **(XBKQ1v2_)** | **Xb-So**  **(XBO1v2_)** | **Xb-Sp**  **(XBP1v2_)** |
| --- | --- | --- | --- | --- | --- | --- | --- | --- | --- | --- |
| ***gcvA*** | 910075 | 2140057 | 810058 | 1730051 | 160014 | 0494 | 1200046 | 2900026 | 2250006 | 720057 |
| ***metR*** | 2560025 | 900025 | 990009 | 2260025 | 160018 | 0498 | 40005 | 350025 | 1960011 | 1770006 |
| ***nhaR*** | 2360049 | 1490049 | 2600062 | 1420042 | 880022 | 1730 | 4190052 | 580020 | 2390037 | 650067 |
| ***cysB*** | 2210009 | 2290025 | 2480042 | 1260022 | 1240064 | 2290 | 3970002 | 120021 | 1300149 | 1210023 |
| ***ynfL*** | 2490048 | 560048 | 2480013 | 1260049 | 1250025 | 2320 | 2370001 | 120048 | 1300122 | 2830026 |
| ***ydhB*** | 2260041 | 1870037 | 50033 | 3080053 | 1300068 | 2497 | 3950022 | 2150005 | 1040057 | 2480041 |
| ***yeiE*** | 2760007 | 2090038 | 2120013 | 840088 | 1550053 | 2880 | 440059 | 1850050 | 2000028 | 270151 |
| ***lrhA*** | 1190023 | 2070078 | 1740082 | 3010081 | 1560016 | 2926 | 440019 | 1850085 | 970022 | 3010058 |
| ***lysR*** | 2510040 | 2220040 | 520040 | 3050018 | 1730005 | 3332 | 270002 | 2140017 | 2260030 | 2940057 |
| ***argP*** | 930010 | 2420055 | 1100076 | 2960034 | 1810030 | 3446 | 2990031 | 1060031 | 1820025 | 1760025 |
| ***leuO*** | 920011 | 2420011 | 1100030 | 2290030 | 1810074 | 3490 | 3000039 | 1990024 | 2270001 | 520040 |
| ***yhaJ*** | 170045 | 110045 | 1310050 | 1940055 | 1930049 | 3863 | 300046 | 790006 | 290073 | 1340046 |
| ***malT*** | 190024 | 130024 | 80023 | 1740023 | 2050022 | 4039 | 370012 | 220023 | 170022 | 110024 |
| ***ilvY*** | 2550011 | 2130052 | 90031 | 1560010 | 2110046 | 4190 | 390040 | 2340011 | 180002 | 740011 |
| ***oxyR*** | 480007 | 1280007 | 2550007 | 1860044 | 2140007 | 4266 | 4140007 | 1110036 | 2530064 | 620007 |
| **-** | 1640023 | 1990058 | 2750024 | 2810025 | 80013 | 0253 | 10009 | 2780038 | 1800039 | 1450059 |
| **-** | 310095 | 1840097 | 1260056 | 1570088 | 130075 | 0382 | 1550004 | 760003 | 1300098 | 2990040 |
| **-** | 2310008 | 1350008 | 1100011 | 2310004 | 2690011 | 1451 | 3000058 | 430004 | 2100015 | 520023 |
| **-** | 910017 | 2150053 | 890006 | 700016 | 1900017 | 3766 | 2020009 | 1660001 | 1300098 | 2990114 |
| **-** | 2380059 | 310077 | 750061 | 1870083 | 480014 | 1128 | 1120007 | 1280065 |  | 930085 |
| **-** | 1540002 | 400002 | 140007 | 2510012 | 1480005 | 2773 |  | 2360005 | 1530003 | 1100004 |
| **-** | 2160021 | 260016 | 1190031 | 1090041 |  |  |  | 2740008 |  | 1620011 |
| **-** |  |  |  |  | 60009 | 0157 |  |  |  |  |
| **-** |  |  |  |  | 2370007 | 4397 |  |  |  |  |
| **-** |  |  |  |  |  | 2330 |  |  |  |  |

Table of genes annotated as toxins in *X. bovienii* genomes as determined by MaGe, listed as the annotated gene. The number designation for each gene is given without the prefixes, which are listed at the top of each column. Dashes in the gene column indicate an unnamed regulator.
