# Supplementary material for: Comparison of Xenorhabdus bovienii bacterial strain genomes reveals diversity in symbiotic functions
Source: BMC Genomics. 2015 Nov 2;16:889. doi: 10.1186/s12864-015-2000-8 (PMC4630870; doi:10.1186/s12864-015-2000-8)
Supplement: Additional file 6: Table S6. — Xenorhabdus bovienii genes predicted to encode two-component regulatory systems. Description: Table of all genes within all X. bovienii strains predicted to encode two-component regulators. (DOC 74 kb) [file 12864_2015_2000_MOESM6_ESM.doc]

**Additional File 6: Table S6. *Xenorhabdus bovienii* genes predicted to encode two-component regulatory systems^a^.**

| **Gene^b^** | **Xb-Sf-FL (XBFFL1v2_)** | **Xb-Sf-FR (XBFFR1v2_)** | **Xb-Sf-MD (XBFM1v2_)** | **Xb-Si**  **(XBI1v2_)** | **Xb-Sj**  **(XBJ2v2_)** | **Xb-Sj-2000**  **(XBJ1_)** | **Xb-Sk-BU**  **(XBKB1v2_)** | **Xb-Sk-CA**  **(XBKQ1v2_)** | **Xb-So**  **(XBO1v2_)** | **Xb-Sp**  **(XBP1v2_)** |
| --- | --- | --- | --- | --- | --- | --- | --- | --- | --- | --- |
| ***malT*** | 190024 | 130024 | 80023 | 1740023 | 2050022 | 4039 | 370012 | 220023 | 170022 | 110024 |
| ***cpxRA*** | 1090026 1090027 | 1130027 1130028 | 1910032 1910033 | 1860003 1860004 | 2390062 2390063 | 4311  4312 | 4140046 4140047 | 2880005 2880006 | 2530021 2530022 | 620048 620049 |
| ***tctD*** | 1190069 1190070 | 2070032 2070033 | 1740029 1740030 | 3010036 3010037 | 1570002 1570003 | 2972  2973 | 210015 210016 | 2570004 2570005 | 2030031 2030032 | 3010002 3010003 |
| ***yfhAK*** | 1310005 1310007 | 1550023 1550025 | 2390005 2390007 | 2040060 2040062 | 1570065 1570067 | 3037  3039 | 2770012 2770014 | 2600046 2600048 | 2110040 2110042 | 2040012 2040014 |
| ***phoBR*** | 1610004 1610005 | 550015  550016 | 600062  600063 | 2280004 2280005 | 870102 870103 | 1687  1688 | 150026 150027 | 580065 580066 | 90020 90021 | 650013 650014 |
| ***glnLG*** | 1640068 1640069 | 1990012 1990013 | 160005  160006 | 2810057 2810058 | 60058  60059 | 209  210 | 1240043 1240044 | 2780002 2780003 | 2550007 2550008 | 1450016 1450017 |
| ***arcAB*** | 170036 2370012 | 110036 1490080 | 1310059 2600024 | 1420070 1940064 | 1820017 1930040 | 3517  3853 | 300037 4190083 | 2630003 2860030 | 290064 2340008 | 1340037 2930050 |
| ***kdpED*** | 1900070 1900071 | 1900025 1900026 | 820104  820105 | 1150047 1150048 | 440138 440139 | 1070  1071 | 100007 100008 | 70005  70006 | 1370022 1370023 | 2520010 2520011 |
| ***uvrY/***  ***barA*** | 330008 1930009 | 650009 1190008 | 860008 1800008 | 2310026 2510006 | 1420014 1860060 | 2676  3656 | 870037 890002 | 2280010 2360011 | 460002 1710072 | 520002 1260008 |
| ***ompR/envZ*** | 2170097 2170098 | 2050015 2050016 | 2050019 2050020 | 2810082 2810083 | 60035  60036 | 0186  0187 | 1240065 1240066 | 1840013 1840014 | 2510053 2510054 | 2200028 2200029 |
| ***phoPQ*** | 2400037 2400038 | 720037  720038 | 320022  320023 | 840044 840045 | 1550012 1550013 | 2835  2836 | 1420013 1420014 | 1850008 1850009 | 2630035 2630036 | 270105 270106 |
| ***rssB*** | 2660011 | 2300019 | 2420035 | 2660028 | 1300007 | 2436 | 180007 | 2150063 | 1300192 | 2720025 |
| ***cheAB*** | 2770018 2770023 | 1260018 1260023 | 2140004 2140009 | 2920041 2920046 | 1000014 1000019 | 1921  1926 | 3820006 3820011 | 2240005 2240010 | 480016 480021 | 3080017 3080022 |
| ***cheY*** | 2770024 | 1260024 | 2140003 | 2920040 | 1000020 | 1927 | 3820005 | 2240004 | 480022 | 3080023 |
| ***yehTU*** | 310086  310087 | 1840086 1840087 | 1260065 1260066 | 1570080 1570081 | 130067 130068 | 0375  0376 | 20062  20063 | 760012 760013 | 1780003 1780004 | 2990047 2990048 |
| ***luxR*** | 310116 | 1840117 | 1260037 | 1570114 | 130092 | 402 | 800002 | 2320025 | 280014 | 2990021 |
| ***uphAB*** | 660003  660004 | 2320005 2320007 | 2330007 2330008 | 1870173 1870174 | 1140029 1610030 | 2122  3090 | 1660007 1660008 | 2730004 2730005 | 1230039 1230044 | 3060021 3060022 |
| ***prpR*** | 740010 | 1560010 | 2730010 | 410002 | 630004 | 1343 | 3980004 | 300043 | 2270020 | 1080006 |
| ***narP*** | 770041 | 1910056 | 320086 | 2640032 | 1330010 | 2592 | 1420078 | 2750012 | 2380011 | 270043 |
| ***baeRS*** | 770074  770075 | 1910023 1910024 | 320054  320055 | 840009 840010 | 1450010 1450011 | 2733  2734 | 1420046 1420047 | 2690006 2690007 | 2380039 2380040 | 270074 270075 |
| ***rcsBC*** | 940012  940013 | 2470012 2470013 | 2280037 2280038 | 400030 400031 | 330015 330016 | 0731  0732 | 1510026 1510027 | 280007 280008 | 2360012 2360013 | 1120032 1120033 |
| ***rcsD*** | 940014 | 2470014 | 2280039 | 400032 | 330017 | 733 | 1510028 | 280009 | 2360014 | 1120034 |
| **RR-1** | 550002 | 830002 | 2100010 | 2920064 | 990005 | 1898 | 3580004 | 740003 | 470001 | 3020002 |
| **RR-2** | 2380032 | 310104 | 750033 | 1870110 | 480007 | 1121 | 570034 | 1280035 | 2660005 | 930060 |
| **HK-1** | 2610007 | 2550013 | 2670003 | 770008 | 1140030 | 2123 | 1930003 | 1950007 |  | 1950015 |
| **RR-3** | 2500011 | 220007 |  |  |  |  | 2970021 |  |  |  |
| **RR-4** |  |  |  | 1620001 | 2740004 | 1569 |  | 2660004 | 2420016 |  |
| **RR-5** |  |  |  |  | 2820006 | 3941 |  |  |  |  |

Table of genes annotated as toxins in *X. bovienii* genomes as determined by MaGe, listed as the annotated gene. The number designation for each gene(s) is given in numerical order without the prefixes, which are listed at the top of each column. HK in gene column designates and unknown histidine kinase, while RR designates an unknown response regulator.
