# Supplementary material for: Comparison of Xenorhabdus bovienii bacterial strain genomes reveals diversity in symbiotic functions
Source: BMC Genomics. 2015 Nov 2;16:889. doi: 10.1186/s12864-015-2000-8 (PMC4630870; doi:10.1186/s12864-015-2000-8)
Supplement: Additional file 7: Table S7. — Annotated toxin genes. Description: Table of all genes within all X. bovienii strains predicted to encode toxin proteins excluding Tc toxin subunits. (DOC 34 kb) [file 12864_2015_2000_MOESM7_ESM.doc]

**Additional File 7: Table S7. Annotated toxin genes^a^.**

| **Gene^b^** | **Gene Products^c^** | **Xb-Sf-FL (XBFFL1v2_)** | **Xb-Sf-FR (XBFFR1v2_)** | **Xb-Sf-MD (XBFM1v2_)** | **Xb-Si**  **(XBI1v2_)** | **Xb-Sj**  **(XBJ2v2_)** | **Xb-Sj-2000**  **(XBJ1_)** | **Xb-Sk-BU**  **(XBKB1v2_)** | **Xb-Sk-CA**  **(XBKQ1v2_)** | **Xb-So**  **(XBO1v2_)** | **Xb-Sp**  **(XBP1v2_)** |
| --- | --- | --- | --- | --- | --- | --- | --- | --- | --- | --- | --- |
| *mcf_Xb_* | Putative Mcf toxin | 1530001 | 1740002 | 2420009 | 2660002 | 1290015 | 2410 | 3530004 | 130003 | 1300170 | 510006 |
| *MARTX_Xb_* | RTX toxin | 1910012 | 1900014 | 820092 | 1150035 | 440015 | 1089 | 110001 | 2030002 | 1370016 | 990035 |
| *prtA* | RTX-like metalloprotease | 910078 | 2140054 | 810055 | 1730048 | 160011 | 0491 | 1200049 | 2900029 | 2250009 | 720054 |
| *xaxAB* | cytotoxin XaxA and transporter XaxB | 2360019  2360018 | 1490019  1490018 | 2600093  2600094 | 1420016  1420015 | 880002  880001 | 1711  1710 | 4190021  4190020 | 580043  580044 | 2390017  2390016 | 650034  650033 |
| *xhlA*  *xhlA2* | Hemolysin | 1640017  360004 | 1990063  1030003 | 2750030  290005 | 2810020  2350006 | 80018 | 0258 | 10018 | 2780047 | 1800045 | 1450065  1130006 |
| *stxA1* | Shiga toxin  A-chain |  |  |  | 2730004 |  |  |  |  |  |  |

^a^Table of genes annotated as toxins in *X. bovienii* genomes as determined by MaGe, listed as the annotated gene. The number designation for each gene is given without the prefixes, which are listed at the top of each column.

^b^Gene annotation for toxin.

^c^Predicted gene product.
