# Supplementary figures and images for: Comparison of Xenorhabdus bovienii bacterial strain genomes reveals diversity in symbiotic functions
Source: BMC Genomics. 2015 Nov 2;16:889. doi: 10.1186/s12864-015-2000-8 (PMC4630870; doi:10.1186/s12864-015-2000-8)

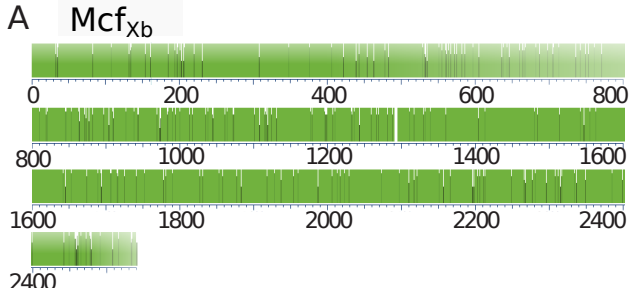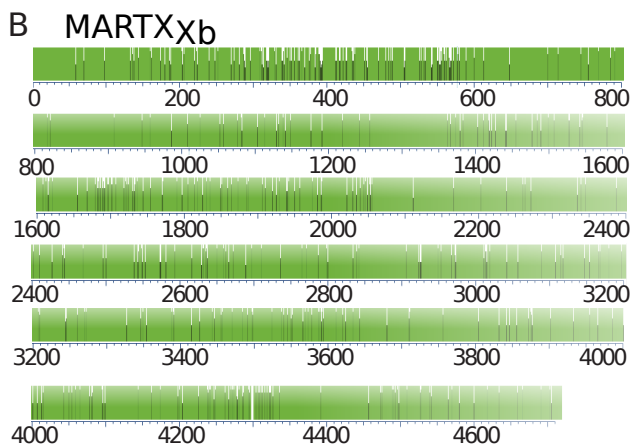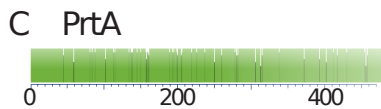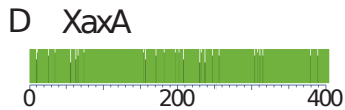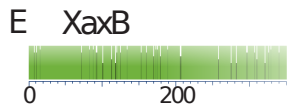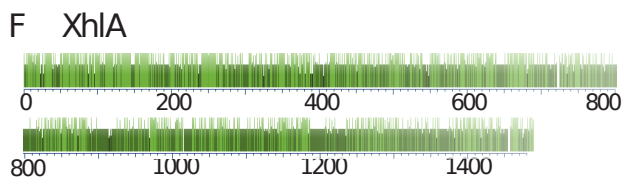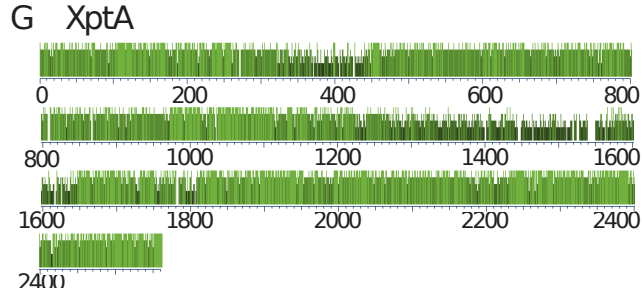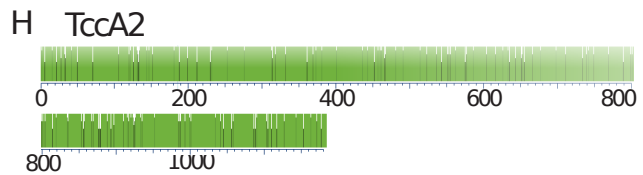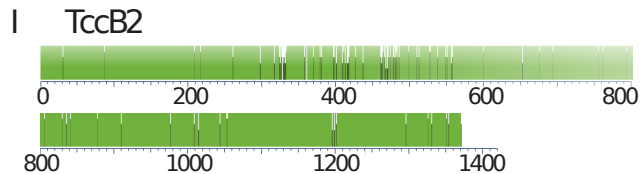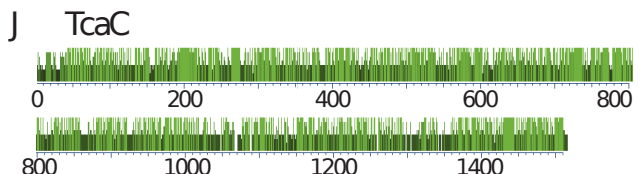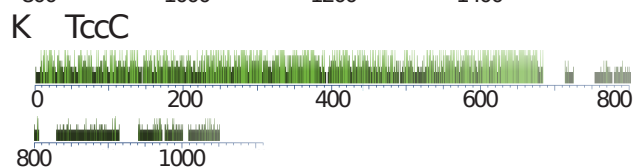

Supplement: Additional file 8: Figure S1. — Distribution of amino acid sequence divergence in proteins. Description: Image of the amino acid sequence divergence among X. bovienii homologs of McfXb, MARTXXb, PrtA, XaxA, XaxB, XhlA/XhlA2, XptA2, TccA2, TccB2, TcaC, and TccC. (PDF 181 kb) [file 12864_2015_2000_MOESM8_ESM.pdf]

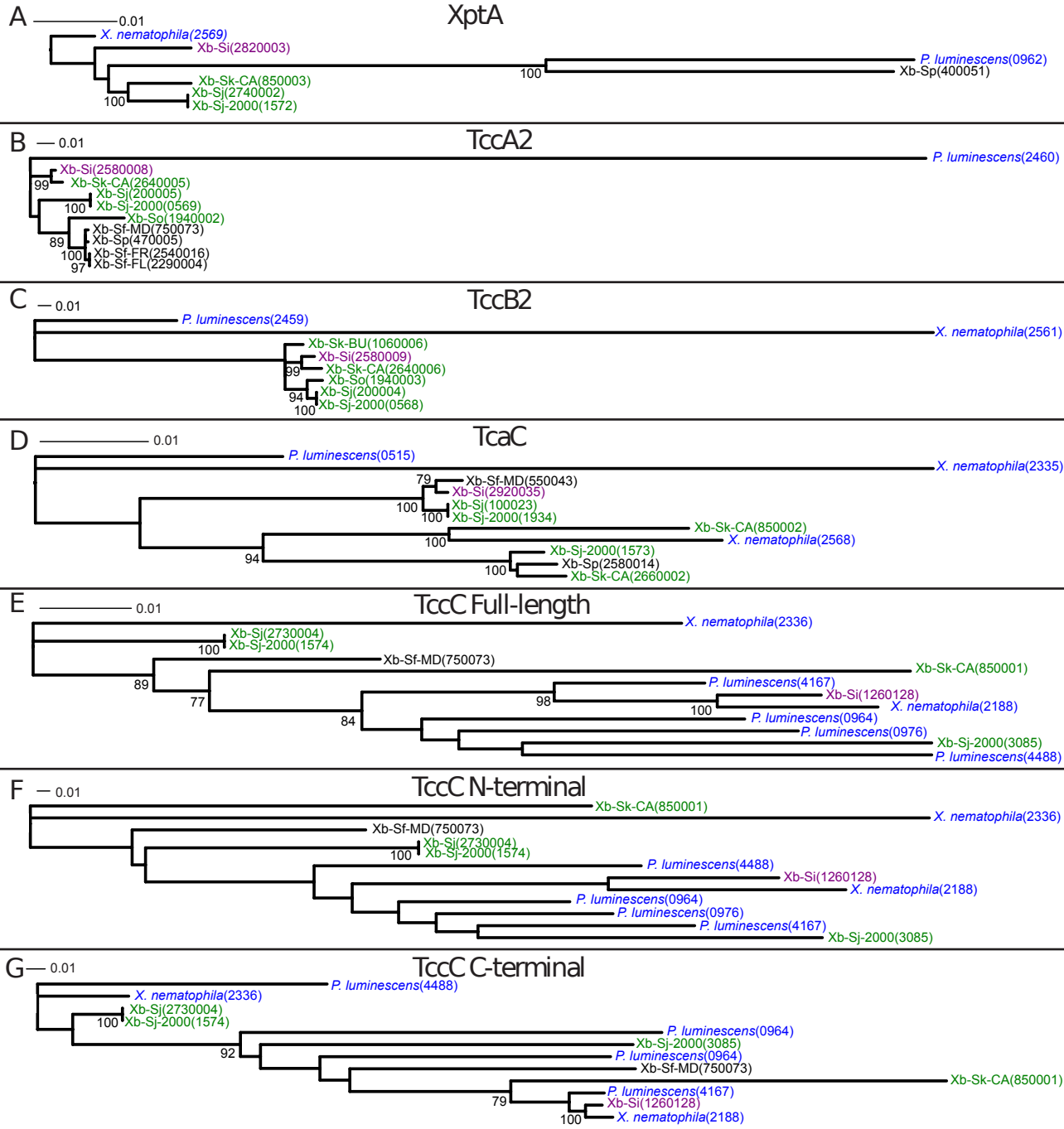

Supplement: Additional file 11: Figure S2. — Phylogenies of TC toxin amino acid sequences. Description: Image of the phylogenies built from amino acid sequences of X. bovienii homologs XptA2, TccA2, TccB2, TcaC, and TccC. (PDF 52 kb) [file 12864_2015_2000_MOESM11_ESM.pdf]
