# Supplementary material for: Comparison of Xenorhabdus bovienii bacterial strain genomes reveals diversity in symbiotic functions
Source: BMC Genomics. 2015 Nov 2;16:889. doi: 10.1186/s12864-015-2000-8 (PMC4630870; doi:10.1186/s12864-015-2000-8)
Supplement: Additional file 9: Table S8. — Best BLASTp hits of Xb-Si putative Shiga toxin. Description: Table of the top 5 BLASTp hits for the putative Shiga toxin from Xb-Si (XbI1v2_2730004). (DOC 30 kb) [file 12864_2015_2000_MOESM9_ESM.doc]

**Additional File 9: Table S8. Best BLASTp hits of Xb-Si putative Shiga toxin^a^.**

| **GenBank Accession Number^b^** | **Description^c^** | **Coverage^d^** | **Identity^e^** | **E-value^f^** |
| --- | --- | --- | --- | --- |
| WP_006035658.1 | Shiga toxin A-chain from *Rickettsiella grylli* | 89% | 35% | 1.0E-37 |
| CAA85366.1 | Shiga-like toxin 1 A-chain from *Escherichia coli* | 98% | 29% | 2.0E-23 |
| WP_001365506.1 | Shiga toxin A-chain from *Escherichia coli* | 98% | 29% | 3.0E-23 |
| CAA85368.1 | Shiga-like toxin 1 A-chain from *Escherichia coli* | 98% | 29% | 3.0E-23 |
| BAC78639.1 | Shiga toxin 1 variant A (*stx1a* in *Escherichia coli*) | 98% | 29% | 3.0E-23 |

^a^Table of Blastp results of the top five hits for XbI1v2_2730004 (Xb-Si putative Shiga Toxin), excluding itself.

^b^The GenBank accession number for each hit.

^c^A short description of the hit based on the provided information in GenBank.

^d^The percentage of the query amino acid sequence that is covered by the hit.

^e^The percentage of the amino acid sequence that is identical between the hit and the query.

^f^Expect (E) value is the number of sequences that you would expect to obtain from the database that match equally well based on chance.
