# Supplementary material for: Comparison of Xenorhabdus bovienii bacterial strain genomes reveals diversity in symbiotic functions
Source: BMC Genomics. 2015 Nov 2;16:889. doi: 10.1186/s12864-015-2000-8 (PMC4630870; doi:10.1186/s12864-015-2000-8)
Supplement: Additional file 10: Table S9. — Tc subunit genes from X. bovienii genomes. Description: Table of all predicted Tc toxin subunit proteins from all X. bovienii genomes, including the intact and fragmented open reading frames. (DOC 128 kb) [file 12864_2015_2000_MOESM10_ESM.doc]

**Additional File 10: Table S9. Tc subunit genes from *X. bovienii* genomes^a^**.

| Genome | ORF^b^ | Gene Annotation^c^ | Subunit Type^d^ |
| --- | --- | --- | --- |
| Xb-Sf-FL | XBFFL1_1440001 | *xptA2/tcdA* | A' |
| Xb-Sf-FL | XBFFL1_1440006 | *xptA2/tcdA* | A' |
| Xb-Sf-FL | XBFFL1_1440007 | *xptA2/tcdA* | A' |
| Xb-Sf-FL | XBFFL1_1440008 | *xptA2/tcdA* | A' |
| Xb-Sf-FL | XBFFL1_2490001 | *xptA2/tcdA* | A' |
| Xb-Sf-FL | XBFFL1_2490003 | *xptA2/tcdA* | A' |
| Xb-Sf-FL | XBFFL1_2290004 | *tccA2* | A |
| Xb-Sf-FL | XBFFL1_2290002 | *tccB2/xptD* | A' |
| Xb-Sf-FL | XBFFL1_2290003 | *tccB2/xptD* | A' |
| Xb-Sf-FL | XBFFL1_1440004 | *xptC/tcaC* | B' |
| Xb-Sf-FL | XBFFL1_1440005 | *xptC/tcaC* | B' |
| Xb-Sf-FL | XBFFL1_2380073 | *tccC6* | C' |
| Xb-Sf-FR | XBFFR1_560001 | *xptA2/tcdA* | A' |
| Xb-Sf-FR | XBFFR1_560003 | *xptA2/tcdA* | A' |
| Xb-Sf-FR | XBFFR1_630033 | *xptA2/tcdA* | A' |
| Xb-Sf-FR | XBFFR1_630034 | *xptA2/tcdA* | A' |
| Xb-Sf-FR | XBFFR1_630035 | *xptA2/tcdA* | A' |
| Xb-Sf-FR | XBFFR1_630040 | *xptA2/tcdA* | A' |
| Xb-Sf-FR | XBFFR1_2540016 | *tccA2* | A |
| Xb-Sf-FR | XBFFR1_2540017 | *tccB2/xptD* | A' |
| Xb-Sf-FR | XBFFR1_2540018 | *tccB2/xptD* | A' |
| Xb-Sf-FR | XBFFR1_630036 | *xptC/tcaC* | B' |
| Xb-Sf-FR | XBFFR1_630037 | *xptC/tcaC* | B' |
| Xb-Sf-FR | XBFFR1_630039 | *tccC1* | C |
| Xb-Sf-MD | XBFM1_1510009 | *xptA2/tcdA* | A' |
| Xb-Sf-MD | XBFM1_1510010 | *xptA2/tcdA* | A' |
| Xb-Sf-MD | XBFM1_1510014 | *xptA2/tcdA* | A' |
| Xb-Sf-MD | XBFM1_1510015 | *xptA2/tcdA* | A' |
| Xb-Sf-MD | XBFM1_1510016 | *xptA2/tcdA* | A' |
| Xb-Sf-MD | XBFM1_900054 | *tccA2* | A |
| Xb-Sf-MD | XBFM1_900052 | *tccB2/xptD* | A' |
| Xb-Sf-MD | XBFM1_900053 | *tccB2/xptD* | A' |
| Xb-Sf-MD | XBFM1_1510012 | *xptC/tcaC* | B' |
| Xb-Sf-MD | XBFM1_1510013 | *xptC/tcaC* | B' |
| Xb-Sf-MD | XBFM1_550043 | *xptC/tcaC* | B |
| Xb-Sf-MD | XBFM1_550044 | *xptC/tcaC* | B' |
| Xb-Sf-MD | XBFM1_750073 | *tccC4* | C |
| Xb-Sf-MD | XBFM1_2330011 | *tccC5* | C' |
| Xb-Sf-MD | XBFM1_2330012 | *tccC5* | C' |
| Xb-Si | XBI1_2820003 | *xptA2/tcdA* | A |
| Xb-Si | XBI1_2580008 | *tccA2* | A |
| Xb-Si | XBI1_2580009 | *tccB2/xptD* | A |
| Xb-Si | XBI1_1670005 | *xptC/tcaC* | B' |
| Xb-Si | XBI1_1680001 | *xptC/tcaC* | B' |
| Xb-Si | XBI1_1690001 | *xptC/tcaC* | B' |
| Xb-Si | XBI1_2830001 | *xptC/tcaC* | B' |
| Xb-Si | XBI1_2840001 | *xptC/tcaC* | B' |
| Xb-Si | XBI1_2920035 | *xptC/tcaC* | B |
| Xb-Si | XBI1_2050001 | *xptC/tcaC* | B' |
| Xb-Si | XBI1_1120001 | *tccC4* | C' |
| Xb-Si | XBI1_1260128 | *tccC1* | C |
| Xb-Si | XBI1_2050001 | *tccC6* | C' |
| Xb-Sj | XBJ2_2740002 | *xptA2/tcdA* | A |
| Xb-Sj | XBJ2_200005 | *tccA2* | A |
| Xb-Sj | XBJ2_200004 | *tccB2/xptD* | A |
| Xb-Sj | XBJ2_2730005 | *xptC/tcaC* | B' |
| Xb-Sj | XBJ2_2740001 | *xptC/tcaC* | B' |
| Xb-Sj | XBJ2_1000023 | *xptC/tcaC* | B |
| Xb-Sj | XBJ2_1610026 | *tccC5* | C' |
| Xb-Sj | XBJ2_2730004 | *tccC7* | C |
| Xb-Sj-2000 | XBJ1_1572 | *xptA2/tcdA* | A |
| Xb-Sj-2000 | XBJ1_1932 | *xptA2/tcdA* | A' |
| Xb-Sj-2000 | XBJ1_1933 | *xptA2/tcdA* | A' |
| Xb-Sj-2000 | XBJ1_0569 | *tccA2* | A |
| Xb-Sj-2000 | XBJ1_0568 | *tccB2/xptD* | A |
| Xb-Sj-2000 | XBJ1_1573 | *xptC/tcaC* | B |
| Xb-Sj-2000 | XBJ1_1934 | *xptC/tcaC* | B |
| Xb-Sj-2000 | XBJ1_2397 | *tcdB* | B' |
| Xb-Sj-2000 | XBJ1_1574 | *tccC5* | C |
| Xb-Sj-2000 | XBJ1_3085 | *tccC7* | C |
| Xb-Sk-BU | XBKB1_700008 | *xptA2/tcdA* | A' |
| Xb-Sk-BU | XBKB1_700009 | *xptA2/tcdA* | A' |
| Xb-Sk-BU | XBKB1_700011 | *xptA2/tcdA* | A' |
| Xb-Sk-BU | XBKB1_700013 | *xptA2/tcdA* | A' |
| Xb-Sk-BU | XBKB1_700014 | *xptA2/tcdA* | A' |
| Xb-Sk-BU | XBKB1_700015 | *xptA2/tcdA* | A' |
| Xb-Sk-BU | XBKB1_1060007 | *tccA2* | A' |
| Xb-Sk-BU | XBKB1_1060008 | *tccA2* | A' |
| Xb-Sk-BU | XBKB1_1060009 | *tccA2* | A' |
| Xb-Sk-BU | XBKB1_1060006 | *tccB2/xptD* | A |
| Xb-Sk-BU | XBKB1_2960024 | *tccC2* | C' |
| Xb-Sk-BU | XBKB1_2960025 | *tccC1* | C' |
| Xb-Sk-CA | XBKQ1_420007 | *xptA2/tcdA* | A' |
| Xb-Sk-CA | XBKQ1_420008 | *xptA2/tcdA* | A' |
| Xb-Sk-CA | XBKQ1_420009 | *xptA2/tcdA* | A' |
| Xb-Sk-CA | XBKQ1_850003 | *xptA2/tcdA* | A |
| Xb-Sk-CA | XBKQ1_2640005 | *tccA2* | A |
| Xb-Sk-CA | XBKQ1_2640006 | *tccB2/xptD* | A |
| Xb-Sk-CA | XBKQ1_2660002 | *xptC/tcaC* | B |
| Xb-Sk-CA | XBKQ1_850002 | *xptC/tcaC* | B |
| Xb-Sk-CA | XBKQ1_850001 | *tccC1* | C |
| Xb-Sk-CA | XBKQ1_850035 | *tccC1* | C' |
| Xb-Sk-CA | XBKQ1_1280067 | *tccC5* | C' |
| Xb-Sk-CA | XBKQ1_2730001 | *tccC5* | C' |
| Xb-So | XBO1_1940002 | *tccA2* | A |
| Xb-So | XBO1_1940003 | *tccB2/xptD* | A |
| Xb-So | XBO1_480026 | *xptC/tcaC* | B' |
| Xb-So | XBO1_1300030 | *tccC1* | C' |
| Xb-Sp | XBP1_400038 | *xptA2/tcdA* | A' |
| Xb-Sp | XBP1_400039 | *xptA2/tcdA* | A' |
| Xb-Sp | XBP1_400040 | *xptA2/tcdA* | A' |
| Xb-Sp | XBP1_400041 | *xptA2/tcdA* | A' |
| Xb-Sp | XBP1_400042 | *xptA2/tcdA* | A' |
| Xb-Sp | XBP1_400051 | *xptA2/tcdA* | A |
| Xb-Sp | XBP1_470005 | *tccA2* | A |
| Xb-Sp | XBP1_470002 | *tccB2/xptD* | A' |
| Xb-Sp | XBP1_470003 | *tccB2/xptD* | A' |
| Xb-Sp | XBP1_470004 | *tccB2/xptD* | A' |
| Xb-Sp | XBP1_400044 | *xptC/tcaC* | B' |
| Xb-Sp | XBP1_400045 | *xptC/tcaC* | B' |
| Xb-Sp | XBP1_400046 | *xptC/tcaC* | B' |
| Xb-Sp | XBP1_2580014 | *xptC/tcaC* | B |
| Xb-Sp | XBP1_400047 | *tccC5* | C' |
| Xb-Sp | XBP1_400048 | *tccC1* | C' |
| Xb-Sp | XBP1_930099 | *tccC2* | C' |
| Xb-Sp | XBP1_2950001 | *tccC4* | C' |
| Xb-Sp | XBP1_3060016 | *tccC5* | C' |
| Xb-Sp | XBP1_3060017 | *tccC5* | C' |

^a^Table of all annotated Tc toxin subunit genes in all X. bovienii genomes (i.e. all 9 draft genomes and finished genome). Grey shading highlights intact genes. Heavier dotted lines delineate different subunits, while full lines delineate different genomes.

^b^Open reading frame (ORF) as labeled in EMBL and GenBank.

^c^Annotation of gene from MaGe. For genes that can have multiple labels both are listed (e.g. *xptC* and *tcaC* are the same genes that were annotated in *X. nematophila* and *P. luminescens* respectively.)

^d^Subunit types that are intact (A, B, or C) or fragments (A’, B’, B’).
